# Preoperative Nutrition-Based Interventions in Children Undergoing Cardiac Surgeries—A Systematic Review and Meta-Analysis

**DOI:** 10.3390/nu18030544

**Published:** 2026-02-06

**Authors:** Agata Stróżyk, Piotr Halicki, Maciej Kołodziej, Andrea Horvath, Michał Buczyński, Radosław Pietrzak

**Affiliations:** 1 Department of Pediatrics, Medical University of Warsaw, 02-091 Warsaw, Poland; 2 Department of Pediatric Cardiology and General Pediatrics, Medical University of Warsaw, 02-091 Warsaw, Poland; piotr.halicki@wum.edu.pl (P.H.);; 3 Department of Cardiothoracic Surgery and Transplantology, Medical University of Warsaw, 02-091 Warsaw, Poland

**Keywords:** preoperative nutrition, congenital heart disease, children, malnutrition, meta-analysis

## Abstract

**Objective**: This systematic review aimed to evaluate the efficacy and safety of preoperative nutrition-based interventions on pre-, intra-, and postoperative outcomes in children undergoing cardiac surgical procedures. **Methods**: CENTRAL, MEDLINE, and EMBASE were systematically searched for interventional and observational studies comparing any nutritional preoperative intervention with a control or alternative strategy in pediatric patients undergoing cardiac surgery, up to July 2025. The main outcome was the postoperative length of stay in the intensive care unit (ICU). The certainty of evidence was assessed using the GRADE approach. **Results**: Nineteen studies were included (8 randomized controlled trials [RCTs], 1 non-randomized trial, and 10 observational studies), evaluating heterogeneous interventions or exposures, including fatty acids, vitamin D supplementation, and structured preoperative nutritional protocols. Two RCTs demonstrated shorter ICU and hospital stays with extended preoperative nutritional support (2 weeks vs. 1 week; n = 40; and 1 month vs. no support; n = 80). Observational data indicated an association between preoperative nutritional support and reduced hospital length of stay (meta-analysis of four studies; n = 278), as well as fewer days to achieve full enteral feeding postoperatively (meta-analysis of three studies; n = 138). No significant difference in postoperative ICU stay was observed between groups (meta-analysis of two studies; n = 175). No intervention-related serious adverse events were reported. The overall certainty of evidence was very low. **Conclusions**: This systematic review provides very low-certainty evidence suggesting that preoperative nutrition-based interventions in children undergoing cardiac surgery are safe and may offer clinical benefits. Substantial heterogeneity across studies underscores the need for well-designed trials and standardized preoperative nutritional protocols. **PROSPERO number:** CRD420251085196.

## 1. Introduction

Despite the significant advances in pediatric cardiac surgeries for congenital heart defects (CHD), preoperative underweight remains common, affecting an estimated 27.4% of children with CHD [1]. A 2019 systematic review reported that malnourished children undergoing surgery for CHD experienced significantly longer hospital and intensive care unit (ICU) stays, as well as mechanical ventilation, compared with well-nourished children [2]. These findings highlight the need for early identification of malnutrition in children preparing for cardiac surgery and for individualized nutritional interventions to improve postoperative outcomes.

In adults, a multimodal, multidisciplinary, and evidence-based perioperative concept called enhanced recovery after surgery (ERAS) is widely recognized, as it has been associated with fewer complications, shorter hospital stay, and faster recovery of gastrointestinal function [3,4]. While in children undergoing abdominal surgery, ERAS protocols have been shown to reduce the length of hospital stay, number of postoperative complications, and readmission rate (systematic review of 12 studies) [5], the overall evidence remains limited.

Several guidelines recommend avoiding prolonged fasting prior to surgery to support adequate preoperative nutrition [6,7,8]. Moreover, current expert recommendations highlight the importance of adequate preoperative caloric intake and enteral nutrition in children undergoing cardiac surgeries [9,10]; however, evidence regarding their effect on perioperative outcomes remains limited, and no standardized preoperative nutritional protocol has been established [11].

The aim of this systematic review was to summarize the evidence on the efficacy and safety of preoperative nutrition-based interventions on pre-, intra-, and postoperative outcomes in children undergoing cardiac surgery.

## 2. Materials and Methods

The protocol of this systematic review was registered in PROSPERO (CRD420251085196). The Preferred Reporting Items for Systematic Reviews and Meta-analysis (PRISMA) guidelines [12] and the Cochrane Handbook for Systematic Review of Interventions, Version 6.4, were followed [13].

### 2.1. Eligibility Criteria

**Type of studies.** Interventional trials and observational studies (including cohort, case-control, and cross-sectional) were included, regardless of setting and country income level.

**Type of population.** The participants were children undergoing any cardiac surgery, regardless of diagnosis (e.g., congenital heart disease) or baseline nutritional status.

**Type of intervention and comparators.** Studies assessing any nutrition-based preoperative intervention, with or without other intervention components, were considered for this review [11]. Although intra- and postoperative interventions were not considered, studies evaluating preoperative nutrition support that continued in the postoperative period were included. For observational studies, the exposure of interest was defined as any preoperative nutrition-based intervention. Studies were included regardless of the comparator used (i.e., no intervention/exposure or an alternative intervention/exposure). We acknowledge that we included highly heterogeneous nutrition strategies, including single-nutrient supplementation, feeding practices, and structured feeding support; however, our aim was to provide a comprehensive synthesis of the available evidence on any form of nutritional support used preoperatively, to inform future guidelines and identify research gaps. Nonetheless, each comparison was reported and discussed separately, allowing for a transparent interpretation of intervention-specific effects.

**Outcomes.** The main outcome was defined as the total length of intensive care unit stay in the post-operative period, reported as the median/mean number of hours or days during the intervention/observation period. Secondary outcomes are outlined in Supplementary Material Table S1.

### 2.2. Search Strategy

A comprehensive search of the Cochrane Central Register of Controlled Trials (CENTRAL, the Cochrane Library), MEDLINE, and EMBASE from their inception to July 2025, following a pre-specified search strategy (for full search strategy, see PROSPERO CRD420251085196) was conducted. No restrictions regarding language, publication date, geography, or study duration were applied. Additionally, manual searches of relevant systematic review articles were conducted. Furthermore, the International Clinical Trial Registry Platform (ICTRP, https://trialsearch.who.int/) was searched manually to identify any ongoing trials.

### 2.3. Data Collection and Management

Data collection and management were prespecified in the protocol registered in PROSPERO (CRD420251085196). Briefly, three reviewers (AS, PH and MK) independently screened the titles, abstracts, and keywords of all studies identified through the search strategy, using EndNote X9 (Version 9.3.3. Philadelphia, PA, USA, The Clarivate Analytics, 2020). Full texts of potentially relevant studies were then retrieved and independently assessed against the eligibility criteria by the reviewers; if required, another review author was consulted (MB, RP and AH).

Two reviewers (AS and PH), using a standardized form, independently extracted pre-specified data in accordance with the registered protocol. To facilitate extraction of study characteristic items, we used ChatGPT-5^®^ Plus (OpenAI^®^, San Francisco, CA, USA) as a supportive tool. The software was applied solely for the initial drafting of study characteristics using a self-developed data extraction form. All outputs were subsequently verified, corrected, and supplemented by the authors to ensure accuracy and completeness. The authors retained full responsibility for data interpretation, synthesis, and final reporting. We contacted Jelveh-Moghaddam et al. to clarify methodological concern; however, no response was received.

### 2.4. Risk of Bias Assessment

The risk of bias for randomized controlled trials (RCTs) and the quality of observational studies were assessed independently by three reviewers (AS, PH and MK). For interventional trials, the risk of bias was assessed using the second version of the Cochrane Collaboration’s risk-of-bias tool for randomized trials (RoB 2) [14]. The assessment was conducted at the study level and aimed to evaluate the ‘intention-to-treat’ effect. Domains 3 and 4 were evaluated for the main outcome or, if not reported, for change in the degree of malnutrition.

For cohort and case-control studies, the study quality was assessed using the Newcastle–Ottawa Scale (NOS). Although key confounders should ideally be prespecified, two stars were assigned for multivariate analyses, including multiple relevant factors, and one star, when only a single relevant confounder was controlled for. Given the predominantly retrospective design of the included studies, it was difficult to confirm that controls had no history of the outcome; moreover, in non-registry-based studies, we cannot exclude the possibility that it was known before study initiation.

### 2.5. Data Analysis

Whenever feasible, data were analyzed using the Review Manager (RevMan) (Version 5.4. The Cochrane Collaboration, London, UK, 2020). For dichotomous outcomes, the risk ratio (RR) or odds ratio (OR) with 95% confidence intervals (Cls) were calculated to compare the intervention/exposure and control groups. For continuous outcomes, the mean difference (MD) between the intervention/exposure and control group was determined. All analyses were performed using a random-effects model. For studies reporting only median values with ranges, the MD and standard deviation (SD) were estimated using the method proposed by Hozo et al. [15]. In one RCT [16], SD was estimated to be zero; therefore, the means and MD could not be calculated. Three trials [16,17,18,19] reported anthropometric measures not as z-scores; therefore, these data were not analyzed.

In cases of substantial heterogeneity in the analyzed populations, interventions, comparators and outcomes, study findings were not pooled and were instead presented narratively. For meta-analyses, heterogeneity was determined using the inconsistency estimation (I^2^, where ≥ 50% indicates substantial heterogeneity) [13]. Although several subgroup analyses were planned, they were not performed because of the small number of eligible studies per comparison.

The certainty of evidence was assessed using the Grading of Recommendations, Assessment, Development, and Evaluation (GRADE) methodology [20] independently, by three authors (AS, MK and PH). We used GRADEpro software (https://www.gradepro.org) to prepare the ‘Summary of findings’ table (GRADEpro GDT, McMaster University and Evidence Prime, 2023).

## 3. Results

### 3.1. Study Selection

**Figure 1:**
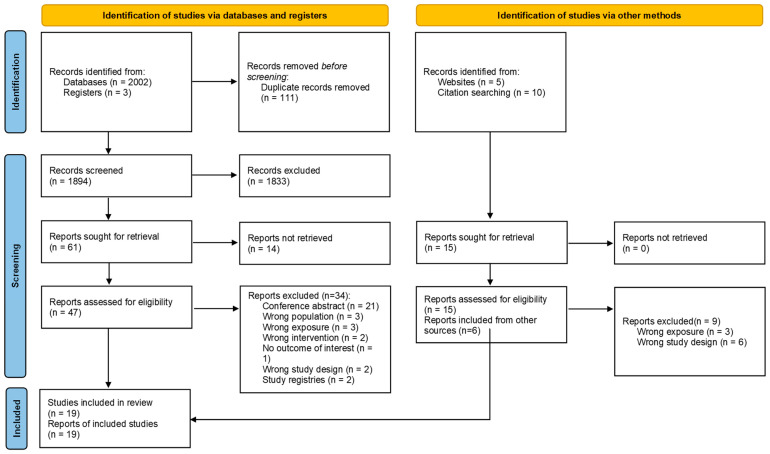
Study selection process (PRISMA 2020 flow diagram).

For the study selection process, see Figure 1. Nineteen studies were included (eight RCTs, one non-randomized trial [non-RCT], nine cohort studies and one case-control study). For studies excluded with reasons, see Supplementary Material Table S2. A search of the ICTRP identified two ongoing RCTs (NCT05457712 and ChiCTR2000031872), both of which are not yet recruiting.

### 3.2. Study Characteristics

#### 3.2.1. Interventional Studies

The characteristics of all included interventional studies are summarized in Supplementary Material Table S3. A total of 402 children were randomized; one non-RCT included 60 participants. Four studies were conducted in North America [18,21,22,23], four in Asia [17,19,24,25], and one in Africa [16].

The majority of trials were single-center (seven RCTs and one non-RCT) [16,17,18,21,22,23,24,25], except for one multi-center RCT [19]. All included interventional trials had two parallel arms. Four studies were double-blinded [17,21,22,23], one was single-blinded [18], two were open-label [16,19] and two did not report blinding [24,25].

Six trials included neonates and infants [16,17,18,19,21,22], whereas three also involved older children (up to 16–18 years of age) [23,24,25]. Cardiac surgery definition varied across trials and included open-heart surgery with cardiopulmonary bypass (three RCTs) [18,22,23], non-restricted ventricular septal defect (two RCTs) [17,19], Blalock–Taussig shunt or aortoplasty for cardiovascular malformations (one RCT) [21], intracardiac repair with cardiopulmonary bypass for tetralogy of Fallot (one RCT) [25], any cardiac surgery (one non-RCT and one RCT), and surgery for non-restricted ventricular septal defect (two RCTs) [16,24].

Nutritional interventions ranged from supplementation with single nutrients (fatty, acids emulsion, vitamin D) to the use human milk fortifier and complex preoperative nutrition protocols. The effects of two fatty acid emulsions were assessed in two trials: enteral docosahexaenoic acid (DHA) in sunflower oil (75 mg/kg of baseline weight/day; in two daily doses) versus sunflower oil alone [21], and an intravenous emulsion containing 50% medium-chain triglycerides (MCTs) and 40% long-chain triglycerides (LCTs) from soybean oil, and 10% fish oil (0.02 g of eicosapentaenoic acid/DHA per ml of total fat; MCT/LCT/fish oil) versus a fully LCT emulsion [22]. Both interventions were continued for 6 and 10 days after surgery, respectively [21,22].

Three trials assessed the effect of high-dose cholecalciferol; however, the administration route, dose, and comparators varied: a single oral dose (10,000 IU/kg up to a maximum of 400,000 IU; one RCT) versus no intervention [25]; enteral supplementation equivalent to 1600 IU/day for children < 1 y or 2400 IU/day for those aged 1 to 17 y; one RCT) versus usual care [23]; and a single intramuscular dose of 300,000 IU (one non-RCT) versus no intervention [24].

One trial in breastfed infants assessed the addition of a human milk fortifier (HMF) to breast milk versus placebo [17]. In one trial, preoperative enteral trophic breast milk feeding (every 3 h, with a total daily volume of 10 mL/kg/day) was compared with no feeding [18]. Moreover, two RCTs assessed the efficacy of different preoperative nutrition protocols. The first trial [16] assessed the effects of a 2-week prehabilitation program using an oral nutrition supplement (ONS) compared with an identical 1-week prehabilitation program. The second trial evaluated the efficacy of an individualized nutritional support protocol initiated one month before surgery, compared with no structured nutritional support plan (Supplementary Material Table S3) [19].

#### 3.2.2. Observational Studies

Eight retrospective cohort studies [26,27,28,29,30,31,32,33], one prospective cohort study [34], and one case-control study [35] were included. The characteristics of the included observational studies are summarized in Supplementary Material Table S4. In total, the studies involved 3902 children. All studies were performed in the United States. Seven studies were single-center [27,30,31,32,33,34,35], and three publications were based on two large multi-center studies [26,28,29].

Eight of the included studies involved children with varied cardiac diagnoses [26,27,28,29,30,31,33,34]; half of these studies involved patients with hypoplastic left heart syndrome (HLHS) or other single-ventricle CHD [28,29,31,34]. In one study, only children with HLHS were eligible for inclusion [32]. Among these studies, five included infants who underwent stage 1 palliation [26,28,29,32,34], and in two of these studies, a Norwood or Norwood variant procedure was performed [29,32]. In four other studies, any cardiac surgery was performed [27,30,31,33]. Additionally, one study included only children undergoing arterial switch operation for the transposition of great arteries [35]. In two of the included studies, only children who were discharged home after surgery were included [27,29].

Exposures varied across the included cohort studies, with most of them (six studies) [26,27,31,32,33,34] assessing any preoperative feeding compared with no feeding; in one of these studies, all children received additionally postoperative feeds [32]. Moreover, two cohort studies assessed the impact of achieving center-recommended pre-operative caloric intake requirements [29], and receiving preoperatively fortified nutrition (≥22 kcal/oz) compared with unfortified nutrition (20 kcal/oz) [30]. Additionally, one case-control study assessed an association between the preoperative feed intake and length of postoperative stay (shorter than 7 days vs. longer than 14 days) [35]. We also identified a propensity score-matched cohort that assessed an association between exclusive human milk feeding and direct breastfeeding and varied intraoperative outcomes [28].

### 3.3. Risk of Bias

**Figure 2:**
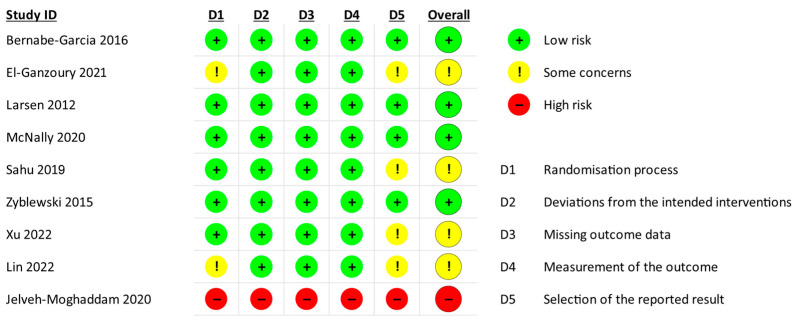
Assessment of risk of bias in randomized controlled trials using the revised Cochrane risk-of-bias tool for RCTs (RoB 2) [16,17,18,19,21,22,23,24,25].

***Interventional trials.*** The risk of bias is reported in Figure 2. The overall risk of bias was assessed as being low in four RCTs [18,21,22,23] and as having some concerns in four RCTs [16,17,19,25]. Two trials [16,17] had an unclear risk of bias related to the randomization process, because allocation sequence concealment was not reported. In four RCTs [16,17,19,25], some concerns were identified regarding the selection of the reported results, mainly due to the lack of a registered or published study protocol. Although one trial [24] was reported as being randomized, the allocation process was not clearly described; therefore, it was judged to be at high risk of non-random assignment. While some RCTs [16,19] were open-label, we did not identify any major deviations from the intended intervention. A sample size calculation was reported in five trials [16,17,19,21,23].

***Observational studies.*** The quality of the cohort studies is summarized in Table 1 and Supplementary Material Table S5. The NOS total score ranged from five (one study) to nine (maximum score, four studies). The weakest item was cohort comparability, based on study design or analysis: only four out of nine studies received two stars (maximum score), whereas three studies did not adjust the outcome for any confounding factors. The NOS total score for one case-control study was five (Supplementary Material Table S6), downgraded by the selection of control groups and lack of control for any confounders.

### 3.4. Effects of Any Modified Fatty Acid Emulsion

#### 3.4.1. Enteral DHA in Sunflower Oil vs. Sunflower Oil Only

Data were reported in one small trial involving 34 children [21].

##### Efficacy

***Length of ICU stay.*** We found a shorter mean duration of ICU stay in the group receiving an enteral DHA in sunflower oil compared with the group with sunflower oil alone (MD = −4.3 days, 95% CI, −5.71 to −2.89) [21].

##### Safety

***Intraoperative adverse events.*** There was no significant difference in the number of children with bleeding during surgery between groups (RR = 0.16; 95% CI, 0.01 to 2.87) [21].

***Postoperative adverse events***

Fewer children with at least one organ dysfunction were observed in the group receiving enteral DHA in sunflower oil compared with the sunflower-oil-only group in both the ITT analysis (RR = 0.45, 95%CI 0.22–0.84, n = 55) and the PP analysis (RR = 0.41, 95%CI 0.23–0.75, n = 34) [21]. Additionally, we found no difference between groups in the proportion of children with:

- Postoperative sepsis and severe sepsis, in both the intention-to-treat (ITT; RR = 0.67, 95%CI, 0.34 to 1.30; and RR = 0.89, 95% CI, 0.51 to 1.56, respectively; n = 55) and per-protocol (PP; RR = 0.38, 95% CI, 0.12 to 1.15; RR = 0.28, 95% CI, 0.03 to 2.26, respectively; n = 34) analyses;
- Any postoperative organ dysfunction, including: respiratory (RR = 0.19; 95% CI, 0.03 to 1.4), cardiovascular (RR = 0.28; 95% CI, 0.03 to 2.26), hematological (RR = 0.16; 95% CI, 0.01 to 2.87), hepatic (RR = 0.37; 95% CI, 0.02 to 8.55), and renal dysfunctions (RR = 0.16; 95% CI, 0.01 to 2.87);
- Vomiting events in the ICU (RR = 1.13, 95% CI, 0.18 to 7.09; n = 34);
- Mortality in either the ITT analysis (RR = 0.21; 0.03 to 1.66; n = 55) or in the PP analysis (RR = 0.37; 0.02 to 8.55; n = 34); none of the deaths were related to DHA administration [21].

#### 3.4.2. Intravenous 50% MCT, 40% LCT and 10% Fish Oil Emulsion (MCT/LCT/Fish Oil) vs. Fully LCT Emulsion

Data were reported in one small study involving 32 subjects [22].

##### Efficacy

Compared with the fully LCT emulsion group, in the MCT/LCT/fish oil emulsion group, we found a shorter:

- Mean duration of the ICU stay (MD = −7.4 days, 95% CI, −10.86 to −3.94);
- Mean duration of mechanical ventilation (MD = −2.1 days, 95% CI, −2.86 to −1.34);
- Mean length of hospital stay (MD = −5.1 days, 95% CI, −8.77 to −1.43) [22].

##### Safety

There was no difference between groups in the proportion of children with postoperative sepsis (RR = 1.0, 95% CI, 0.41 to 2.45) [22].

#### 3.4.3. High-Dose Cholecalciferol vs. Usual Care/No Intervention

Data were reported in two RCTs (n = 101) and one non-randomized trial (n = 60).

##### Efficacy

***Length of ICU stay.*** A meta-analysis of two RCTs showed no difference in the mean length of ICU stay between the high-dose cholecalciferol group and any control group (usual care or no supplementation) (MD = −2.25 h, 95% CI, −5.83 to 1.32; I2 = 97%; n = 101; Supplementary Material Figure S1) [23,25]. However, this evidence should be interpreted with caution due to differences in dosing between the two trials and the very low certainty of evidence.

***Length of mechanical ventilation.*** In a meta-analysis of two RCTs [23,25], no difference was found in the mean length of mechanical ventilation between the high-dose cholecalciferol group and any control group (MD = −22.02 h, 95% CI, −58.86 to 14.82; I2 = 92%; n = 101; Supplementary Material Figure S2); however, the certainty of evidence was very low. Moreover, in one of these trials [23], a shorter mean length of initial mechanical ventilation was reported in the enteral high-dose cholecalciferol group compared with the usual care group (MD = −34.0 h; 95% CI, −38.87 to −29.13; n = 41).

Additionally, in one non-RCT [24], we found a longer duration of mechanical ventilation in children who received a single intramuscular injection of cholecalciferol (300 000 IU) three days before the surgery, compared with the no intervention group (MD = −6.75 h, 95% CI, −11.2 to −2.3; n = 60). However, these findings are limited by a high risk of bias.

***Length of hospital stay.*** In one RCT [23], a shorter mean length of hospital stay was observed in the enteral high-dose cholecalciferol group compared with the usual care group (MD = −4.75 days, 95%CI, −6.47 to −3.03, n = 41).

***Administration of inotropes.*** This outcome was reported in one RCT [23], with no difference in the proportion of children receiving catecholamines between the enteral high-dose cholecalciferol group and the usual care group (RR = 0.7, 95% CI, 0.43 to 1.13; n = 41).

##### Safety

***Adverse events.*** Data were reported in two RCTs. In one trial [23], there was no difference between the enteral high-dose cholecalciferol group and the usual care group in any of the reported adverse events: hypocalcemia; post-operative, acute renal failure requiring dialysis; intraoperative and post-operative day 1 hypercalciuria; and transient (<24 h) hypercalcemia during pediatric ICU admission and positive post-operative cultures (n = 41). In the other RCT [25], there was no difference in the proportion of the participants with low cardiac output syndrome (RR = 0.67, 95% CI, 0.21 to 2.13) or junctional ectopic tachycardia (RR = 1.5, 95% CI, 0.27 to 8.34) between the oral high-dose cholecalciferol group and the no intervention group (n = 60). Additionally, no neurological deficit was identified in any of the participants.

***Mortality.*** This outcome was reported in two RCTs [23,25], with no deaths observed (n = 101).

##### Compliance

Compliance was reported in one RCT [23], with a high median compliance rate (94%, IQR 77 to 100, n = 41). There was no difference between the enteral high-dose cholecalciferol group and the usual care group in the mean compliance rate (MD = −8.6%, 95% CI −21.7 to 4.5) or in the number of doses received by participants (MD= −2 doses, 95% CI −26 to 24 doses).

#### 3.4.4. Effect of Human Milk Fortifier vs. Placebo in Breastfed Infants

Data were reported in one trial (n = 58) [17]. All preoperative outcomes were reported one month after nutritional intervention.

##### Efficacy

***Preoperative laboratory markers of malnutrition.*** Higher preoperative levels of albumin (MD = 5.70 g/L, 95% CI, 3.25, 8.15) and prealbumin (MD = 49.4 mg/L, 95% CI, 34.57 to 64.23) were observed one month after the intervention in the HMF group compared with the placebo group, with no difference in preoperative hemoglobin levels between groups (MD = 3.3 g/L, 95% CI, −5.77 to 12.37) [17].

***Preoperative validated malnutrition risk score.*** A lower mean preoperative STRONG Kids Score was observed in the HMF group compared with the placebo group (MD = −1.9, 95% CI, −2.29 to −1.51) [17].

##### Safety

We found no difference between the study groups in the number of children with preoperative pneumonia (RR = 0.75, 95% CI, 0.18 to 3.06), liver insufficiency (RR = 0.5, 95% CI, 0.05 to 5.21), jaundice (RR = 0.5, 95% CI, 0.05 to 5.21), or feeding intolerance (RR = 3.0, 95% CI, 0.33 to 27.18) [17]. Preoperative necrotizing enterocolitis (NEC), gastrointestinal bleeding, and death were not reported in any child.

#### 3.4.5. Effects of Preoperative Nutrition-Based Protocols

##### Efficacy of Preoperative Trophic Breast Milk Feeds

Data were reported in one small trial (n = 27) [18].

Efficacy

***Postoperative feeding tolerance.*** We found no difference between groups in the number of children who postoperatively required a formula change due to feeding intolerance (RR = 1.39, 95% CI 0.69 to 2.82), were dependent on a nasogastric tube at hospital discharge (RR = 1.06, 95% CI, 0.54 to 2.09), or received exclusive breast milk feeds at discharge (RR = 0.93, 95% CI, 0.29 to 2.97) [18].

Safety

One trial found no preoperative adverse events associated with preoperative trophic breast milk feeds [18]. Moreover, no differences between groups were observed in the proportion of children with postoperative NEC (RR = 1.86, 95% CI, 0.19 to 18.13); in those who required gastroesophageal reflux medication at discharge (RR = 1.24, 95% CI, 0.81 to 1.89); and in the postoperative mortality rate (RR = 4.67, 95% CI, 0.24 to 88.96), with no deaths reported in the preoperative trophic breast milk feeds group [18].

##### A 2-Week vs. 1-Week Preoperative Nutrition Support

Data were reported only in one small trial (n = 40) [16].

Efficacy of the 2-week vs. 1-week preoperative nutrition support

Compared with the 1-week prehabilitation group, in the 2-week prehabilitation group, we found:

- A shorter mean length of ICU stay (MD = 36.5 h, 95% CI, −44.61 to −28.39) [16];
- A shorter mean hospital length of stay (MD = −40.9 days, 95%CI, −65.26 to −16.54) [16];
- A shorter mean duration of postoperative mechanical ventilation was found (MD = −14.0 h, 95% CI, −17.95 to −10.05) [16];
- A higher mean postoperative feeding volume intake, measured before discharge (MD = 7.53 mL/feed, 95%CI, 0.99 to 14.07) [16].

However, no difference between groups was found for:

- Other feeding-related outcomes, including: day of enteral feeding initiation (RR = 1.33, 95% CI, 0.88 to 2.0 3, and RR = 1.00, 95% CI, 0.34 to 2.93, for day 1 and 2, respectively), route of feeding (oral: RR = 1.42, 95% CI, 0.95 to 2.12, vs. oral and nasogastric tube: RR = 0.38, 95% CI, 0.12 to 1.21), and feeding frequency (every 2 h: RR = 1.19, 95% CI, 0.93 to 1.51, vs. every 3 h: RR = 0.25, 95% CI, 0.03 to 2.05) [16];
- The proportion of children with successful extubation (RR = 1.05, 95% CI, 0.92 to 1.2), early extubation (≤48 h) (RR = 1.2, 95% CI, 0.9 to 1.61), late extubation (>48 h) (RR = 0.33, 95% CI, 0.04 to 2.94), and reintubation (RR = 0.5, 95%CI, 0.05 to 5.08) [16].

***Change in degree of malnutrition***

*Preoperative anthropometric measures.* The authors reported a higher median weight-for-age z-score in the 2-week prehabilitation group compared with the 1-week prehabilitation group, based on the *p*-value (*p* = 0.001), with no difference in height-for age-z-score between groups (*p* = 0.16).

*Postoperative anthropometric measures.* A higher mean weight-for-age and height-for-age z-scores were observed in the 2-week prehabilitation group compared with the 1-week prehabilitation group (MD = 2.50, 95% CI, 2.15 to 2.85; and MD = 1.00, 95% CI, 0.69 to 1.31, respectively).

*Laboratory markers of malnutrition.* No differences between study groups were observed in albumin and hemoglobin levels measured at discharge (MD = 0.00 gm, 95% CI, −0.16 to 0.16; MD = −0.08 g/dL, 95% CI, −0.92 to 0.76; respectively) [16].

Safety of the 2-week vs. 1-week preoperative nutrition support

***Adverse events.*** Only the proportion of children with nosocomial sepsis was reported; however, no difference was observed between the study groups (RR = 0.2, 95% CI, 0.01 to 3.92) [16].

***Feeding-related adverse events.*** We found no difference in the proportion of children with any feeding-related adverse events between the study groups, including abdominal distension (RR = 0.43, 95% CI, 0.13 to 1.43), increased gastric residue (RR = 0.14, 95% CI, 0.01 to 2.6), vomiting (RR = 0.33, 95% CI, 0.01 to 7.72), diarrhea (RR = 0.33, 95% CI, 0.01 to 7.72), and hematemesis (RR = 0.2, 95% CI, 0.01 to 3.92) [16].

##### Efficacy of a 1-Month Preoperative Nutrition Support vs. No Support

One trial involving 80 children was identified [19].

***Length of ICU and hospital stay***. A shorter mean length of ICU stay and time to discharge were observed in the 1-month preoperative nutrition support group compared with the no-support group (MD = −1.40 days, 95% CI, −1.93 to −0.87; and MD = −3.30 days, 95% CI, −5.09 to −1.51, respectively) [19].

***Preoperative degree of malnutrition***

*Validated malnutrition risk score.* We found a significantly lower mean preoperative STRONG Kids Score in the 1-month preoperative nutrition support group compared with the no-support group (MD = −0.50, 95% CI, −0.72 to −0.28) [19].

*Laboratory markers of malnutrition.* We found a higher level of preoperative albumin, prealbumin and hemoglobin levels in the month preoperative nutrition support group compared with the no-support group (MD = 2.60 g/L, 95% CI, 1.45, 3.75; MD = 10.30 mg/L, 95% CI, 1.03 to 19.57; and MD = 4.20 g/L, 95% CI, 1.14 to 7.26, respectively) [19].

##### Effects of Any Preoperative Feeding vs. No Feeding

Data were reported in six cohort studies and one case-control study.

Effectiveness

***Length of ICU.*** In a meta-analysis of two cohort studies [31,32], we found no difference in the mean postoperative length of ICU stay between the group receiving any preoperative feeding and the no-feeding group (MD = −3.56 days, 95% CI, −7.22 to 0.11, I2 = 23%, n = 175; Supplementary Material Figure S3); however, the certainty of the evidence is very low. Moreover, in one cohort study [33], the mean length of ICU stay did not differ between the standardized preoperative feeding protocol group and the no-feeding protocol group, based on the reported *p*-value (*p* = 0.14; n = 51).

**Figure 3:**
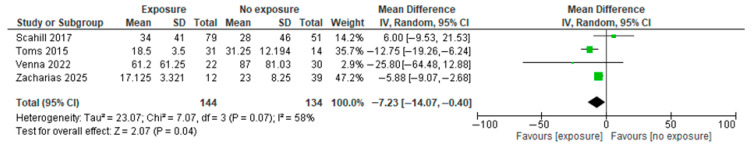
Association between any preoperative feeding and length of hospital stay [31,32,33,34].

***Length of hospital stay.*** A meta-analysis of four cohort studies [31,32,33,34] found a shorter mean length of hospital stay in the group receiving any preoperative feeding compared with the no-feeding group (MD = −7.23 days, 95% CI, −14.07–0.4, I2 = 58%, n = 278; Figure 3); however, the certainty of evidence is very low.

***Duration of mechanical ventilation.*** In one cohort study [31], no difference in the mean duration of mechanical ventilation was observed between the group receiving any preoperative feeding and the no-feeding group (MD = −2.0 days, 95% CI, −7.6 to 3.6, n = 130). In another cohort study [32], a lower mean duration of mechanical ventilation was observed in the group receiving preoperative trophic feeds compared with no feeds (MD = −2.78 days, 95% CI, −3.84 to −1.71, n = 45).

***Change in degree of malnutrition***

*Anthropometric measures at discharge*. In one cohort study [31], no difference in the mean change in weight-for-age z-score from hospital admission to discharge was observed between the group receiving any preoperative feeding and the no-feeding group (MD = −0.04, 95% CI, −0.73 to 0.65, n = 130). In another cohort study [33], no differences were reported in median weight-for-age, length-for-age, and head circumference-for-age at discharge between the standardized preoperative feeding protocol group and the no-feeding protocol group (*p* = 0.52, 0.92 and 0.93, respectively; n = 51).

*Postoperative laboratory markers.* In one study [32], the lowest albumin level in the first 72 h after surgery was higher in the group receiving preoperative trophic feeds compared with no feeds (MD = 0.23, 95% CI, 0.16 to 0.29, n = 45).

**Figure 4:**
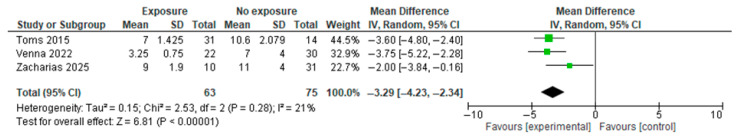
Association between any preoperative feeding and mean number of days to achieve full feeds postoperatively [32,33,34].

***Mean number of days to achieve full feeds postoperatively*.** A meta-analysis of three cohort studies [32,33,34] showed a lower mean number of days required to achieve full postoperative feeds in the group receiving any preoperative feeding compared with the no-feeding group (MD = −3.29, 95% CI, −4.23 to −2.34, n = 138, I2 = 21%; Figure 4). However, the certainty of evidence is very low. One of these studies [32] also reported a shorter time to the first postoperative feed in the group receiving preoperative trophic feeds compared with the nothing-by-mouth group (MD = −0.75 day, 95% CI, −1.28 to −0.22, n = 45). Additionally, one study [34] found no association between preoperative feeding and the need for G-tube placement within the first postoperative year (OR = 0.29, 95% CI, 0.08 to 1.07, n = 52).

Moreover, one study [27] reported a higher proportion of children with full post-operative feeding at discharge in the group receiving any preoperative feeding (OR 2.78, 95% CI 1.48–5.24, n = 235), including those receiving >20 mL/kg/day (adjusted OR = 2.92, 95% CI, 1.28 to 6.69) and ≤20 mL/kg/day (adjusted OR = 2.25, 95% CI, 1.06 to 4.79), compared with the group without any preoperative oral feeding.

In one case-control study [35], a higher proportion of children fed preoperatively (the majority received ad libitum oral feeds, accounting for their full caloric intake) was observed in the short-stay group (<7 days) compared with the long stay group (<14 days) (OR = 8.8, 95% CI, 2.57 to 30.18, n = 57; Supplementary Material Figure S4).

Safety

***Severe cardiac surgery-associated acute kidney injury.*** In a secondary analysis of the NEPHRON multicenter cohort [26], the authors reported 52% lower odds of severe cardiac surgery-associated acute kidney injury in the group receiving preoperative feeding (adjusted OR = 0.48; 95% CI, 0.27 to 0.86, n = 347).

**Figure 5:**
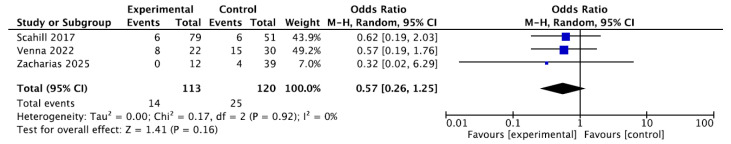
Association between any preoperative feeding and risk of necrotizing enterocolitis [31,33,34].

***NEC.*** In a meta-analysis of three cohort studies [31,33,34], we found no association between the proportion of children with NEC and previous exposure to preoperative feeding (OR = 0.69, 95%CI, 0.26 to 1.82, n = 232; Figure 5). However, the certainty of the evidence is very low. Additionally, in one of these studies [31], no association was observed between preoperative enteral feeding and NEC (OR = 0.62, CI 0.19–2.0, n = 130). Moreover, no association was found between the intake of feeding volumes exceeding >20 mL/kg/day and an increased risk of NEC (OR = 4.04, 95% CI, 0.49 to 33.3) [31]. One study [34] also reported no difference in the risk of preoperative, stage 1 or stage 2 NEC between children who were fed and not fed preoperatively (n = 42).

***Postoperative infection rate.*** One cohort study [32] found no association between the postoperative infection rate and receipt of preoperative trophic feeds compared with nothing-by-mouth (OR = 1.07, 95% CI, 0.23; 4.94, n = 45).

***Mortality.*** A meta-analysis of two cohort studies [32,34] showed no difference in the number of deaths between the group receiving any preoperative feeding and the no-feeding group (OR = 0.65, 95% CI, 0.25 to 1.69, n = 97, I2 = 0%; Supplementary Material Figure S5), but the certainty of evidence is very low.

##### Effectiveness of Meeting Versus Not Meeting Center-Recommended Preoperative Caloric Intake Requirements (Cohort Study)

In one cohort study [29], failure to achieve center-recommended preoperative caloric intake requirements at the last visit before a bidirectional Glenn procedure was associated with a longer post-BDG hospital length of stay (adjusted HR = 1.81, 95% CI, 1.13 to 2.87, n = 160).

##### Preoperative Fortified vs. Unfortified Nutrition

Data were reported in one retrospective cohort study (n = 84) [30].

Effectiveness

***Length of ICU and hospital stay.*** The authors reported a shorter length of ICU stay (MD = 1.00 days, 95% CI, 0.76 to 1.24) and hospital stay (MD = 0.38 day, 95% CI 0.09 to 0.66) in children receiving unfortified nutrition (20 kcal/oz) compared with infants receiving fortified nutrition (≥22 kcal/oz) [30].

***Duration of mechanical ventilation.*** No difference in the duration of mechanical intubation was observed between groups [30].

***Postoperative anthropometric measures.*** Based on *p* values only, the authors reported lower postoperative weight-for-age percentiles in the group receiving fortified nutrition at 2, 5 and 10 years of age, but not at the first 30 and 31–60 days after surgery, compared with children receiving unfortified nutrition [30]. BMI-for-age percentiles were also higher at 5 and 10 years of age (*p* = 0.04 [n = 52] and 0.02 [n = 41], respectively) in the group receiving unfortified nutrition compared with the fortified group (*p* = 0.045 and 0.02, respectively), but not at 2 years of age. However, no difference between groups was observed in the change in weight-for-age after repair at 30 days (n = 23) or 60 days after surgery.

Safety

No difference in the risk of surgical site infection was observed between groups [30].

#### 3.4.6. Preoperative Exclusive Human Milk and Direct Breastfeeding

In one propensity-score-matched cohort [28], involving 2491 participants, the association between preoperative human milk feeding and direct breastfeeding and several outcomes of interest were assessed. Some evidence suggested that exclusive human milk feeding and any direct breastfeeding were associated with a shorter hospital length of stay (data are summarized briefly in the Supplementary Material Table S7).

### 3.5. Quality of Evidence

In all meta-analyses, the certainty of evidence was very low (Supplementary Material Table S8).

## 4. Discussion

### 4.1. Summary

This systematic review summarized evidence from 9 interventional and 10 observational studies on the efficacy/effectiveness and safety of preoperative nutrition intervention on pre-, intra- and postoperative outcomes in children undergoing cardiac surgery. Half of the included trials had some methodological concerns. The quality of observational studies ranged from moderate to high.

An overall summary of findings is presented in Table 2. The intervention/exposures across studies were heterogenous and included parenteral fatty acid emulsions, enteral high-dose vitamin D, HMF, trophic breast milk feeds and various preoperative nutrition support protocols. The main finding of this systematic review is the provision of evidence from a limited number of RCTs and observational studies, suggesting the overall safety of different preoperative nutrition interventions in children undergoing cardiac surgery. While the magnitude and type of benefits varied across interventions, most preoperative nutrition strategies were associated with some favorable outcomes. Enteral DHA in sunflower oil and intravenous MCT/LCT/fish oil emulsion were associated with a shorter ICU length of stay in two small trials. Similarly, shorter ICU and hospital stays were observed with 2-week and 1-month preoperative nutrition support in two RCTs. Observational studies demonstrated an association between any preoperative nutrition support and a shorter hospital length of stay (meta-analysis of four studies), as well as fewer days required to achieve full postoperative feeding (meta-analysis of three studies). However, the findings of this systematic review are limited by the very low certainty of the available evidence.

Intraoperative outcomes and changes in the degree of malnutrition were rarely and poorly reported. Compliance was reported in only one RCT. Postoperative health-related quality of life and readmission rate were not reported in any of the included trials.

### 4.2. Comparison with Other Systematic Reviews

A 2019 systematic review of five cohort studies focused on ductal-dependent lesions [36] reported insufficient evidence for the benefit of preoperative feeding on postoperative outcomes, highlighting small sample sizes and variable protocols. Similarly, Bell et al. (2022) [37] conducted a PRISMA-guided systematic review in neonates and infants awaiting cardiac surgery and found no association between preoperative enteral feeding and NEC, nor with any secondary outcomes such as length of hospital stay and tube-assisted feeding at discharge. However, their dataset was based mainly on observational studies (eight of nine studies), which limits robustness of these findings.

Additionally, in 2022, the Neonatal Cardiac Care Collaborative (NeoC3) synthesized the available evidence across six domains (energy and protein needs, enteral nutrition, feeding practices, parenteral nutrition, and outcomes) and developed consensus statements for infants < 6 months of age with CHD [38]. This document highlighted wide practice variation, frequent reliance on low-level evidence, and the absence of adequately powered RCTs—particularly regarding standardized preoperative feeding advancement and the role of lipid formulations—underscoring the need for multicenter trials and quality-improvement frameworks.

Recently, a non-systematic review on perioperative nutrition support in children with congenital heart disease and heart failure emphasized the need for the implementation of pragmatic, institution-level pathways [39]. Proposed nutrition protocols included standardized preoperative feeding algorithms, liberalization of fasting within ERAS-consistent care, coordinated escalation of enteral versus parenteral strategies, and integration of macronutrient and protein targets and lipid considerations alongside micronutrient supplementation. Although complementary in scope to our work, this review does not provide quantitative pooling or CHD-only stratification and therefore could not estimate effect sizes for preoperative interventions; nevertheless, it aligns with our conclusions regarding the paucity of high-quality trials and the need for standardized preoperative nutritional protocols.

Consistent with our findings, Szentirmay et al. [40], in a recent nonsystematic review, highlighted feasibility and safety signals alongside substantial heterogeneity and limited-quality evidence for preoperative enteral nutrition in neonates with CHD. However, their scope did not address broader preoperative nutrition strategies (e.g., fortification, structured nutrition support, or parenteral approaches) or older infants, which were evaluated in our systematic review.

In contrast to these previous works, our review summarizes a broader spectrum of preoperative nutrition-based interventions, including structured nutrition support programs, feed fortification, lipid emulsions, and short-term prehabilitation elements, and quantitatively explores postoperative endpoints, such as length of stay and time to full feeds. While our pooled estimates suggest potential benefits without major safety signals, the certainty of the evidence was very low and substantial heterogeneity was observed across populations, interventions, and outcome definitions. Overall, our findings remain consistent with the limitations identified by previous reviews [37,38] and reinforce the need for standardized, multicenter trials.

### 4.3. Strengths and Limitations

A key strength of this review is its rigorous methodology, with clearly defined eligibility criteria and clinically relevant outcome measures, and the use of recommended tools for assessing the risk of bias for RCTs and the quality of observational studies. This strengthens the reliability and robustness of these findings. Moreover, the inclusion of both interventional trials and observational studies, regardless of any language or setting restrictions, allows for a broader synthesis and assessment of the impact of different nutritional interventions. We are confident that this systematic review summarizes all currently available evidence on preoperative nutrition interventions on perioperative outcomes.

Additionally, the inclusion of a large number of children undergoing various cardiac surgeries for different cardiac conditions and from different regions enhance the generalizability of the results. However, due to limited sample sizes for individual comparison, we did not perform subgroup analyses, which limits the applicability of the findings to specific diseases or types of surgery.

Nonetheless, several methodological limitations should be considered. A major limitation of this systematic review is the heterogeneity of reported interventions/exposures and outcome measures, which complicates comparison and data pooling and reduces the ability to draw firm conclusions across studies. Many studies reported only within-group differences and statistical significance based solely on *p*-values, without presenting the preferred effect measures. To improve the interpretability of the results, between-group differences were calculated whenever feasible.

Although, the overall quality of the included studies was moderate or high. We need to emphasize that most evidence regarding preoperative nutrition support protocols was derived from observational studies, which precludes any causal inferences and limits the strength of the conclusions. A key limitation of the included observational studies was the lack of adjustment for potential confounders. Confounding may lead to spurious associations when a true causal relationship does not exist; therefore, these findings should be interpreted cautiously [41]. Potential factors affecting outcome or exposure should be carefully considered during the development of future studies.

For all conducted meta-analyses, the certainty of evidence is very low, and it was downgraded mainly because of substantial inconsistency and indirectness, and for observational studies’ methodological limitations. Given the substantial heterogeneity observed in most meta-analysis, these results should be treated as exploratory and hypothesis-generating, rather than immediately clinically relevant.

Another important limitation of most included studies is the small sample size, with evidence largely being derived from single-center cohorts. Only two registry-based observational studies included a substantial number of participants (ranging from 347 to 2491 participants) [26,28]. Although limited by their observational design, multi-center patient registries represent a promising approach to achieving an adequate sample size, particularly for the assessment of long-term efficacy and safety outcomes [42].

There is currently no standardized core outcome set for assessing the efficacy of preoperative nutrition interventions; therefore, we selected outcomes which we considered to be clinically important. The length of ICU stay was chosen as a main outcome because it is commonly reported, and in our opinion, highly clinically relevant, as it reflects the postoperative outcomes and resource utilization. Nonetheless, we acknowledge that it may be affected by center-specific discharge practices, postoperative care pathways, and baseline patient severity and nutritional status, which should be addressed in future trials.

## 5. Conclusions

This systematic review provides very low-certainty evidence suggesting that preoperative nutrition-based interventions may be safe and potentially beneficial in children undergoing cardiac surgery. The main limitations are the variability of the included interventions and outcomes, and the limited certainty of evidence, both of which hinder the formulation of consistent conclusions regarding the effectiveness of preoperative nutrition strategies. Although the current evidence does not allow us to draw any conclusions regarding the superiority of any specific intervention, findings from individual RCTs and observational studies suggest that structured nutrition protocols may be promising. However, further research is required to establish standardized preoperative nutrition protocols with confirmed efficacy and safety in well-designed, adequately powered trials. Additionally, these findings highlight the lack of robust evidence regarding the efficacy of different fatty acid formulations used in parental nutrition, which, given their clinical importance, should be addressed in future RCTs. The development of a core outcomes set for trials assessing nutritional intervention in pediatric patients undergoing cardiac surgeries would further support the adequate selection and standardized reporting of clinically relevant outcomes.

## Figures and Tables

**Table 1:** Summary of assessment of observational studies quality, using the Newcastle–Ottawa Scale (NOS).

Autor (Year)	Selection (0–4)	Comparability (0–2)	Outcome (0–3)	Overall (0–9)
Bertrandt 2024 [26]	★★★	★	★★★	7
Zacharias 2025 [33]	★		★★★	5
Venna 2022 [34]	★★★★	★	★★★	9
Toms 2015 [32]	★★★		★★★	6
Scahill 2017 [31]	★★★★	★	★★★	9
Murray 2025 [30]	★★★	★	★★★	7
Menon 2013 [29]	★★★★	★	★★★	9
Elgersma 2023 [28]	★★★★	★	★★★	8
Dabbagh 2025 [27]	★★★★	★	★★★	9

**Table 2:** Summary of findings.

Intervention/Exposure vs. Comparator	Number of Studies and Participants (n)	Efficacy/Effectiveness	Safety
Intravenous 50% MCT, 40% LCT and 10% fish oil vs fully LCT emulsion [22]	1 RCT, n = 32	↓ ICU stay ↓ Hospital stay ↓ MV	Sepsis—ND
Enteral DHA in sunflower oil vs sunflower oil only [21]	1 RCT, n = 34	↓ ICU stay	Intraoperative bleeding—ND Postoperative sepsis—ND Bleeding—ND Severe sepsis—ND Organ dysfunctions—ND Vomiting at ICU—ND Mortality—ND
High-dose vitamin D vs usual care/no intervention [23,24,25]	2 RCTs, n = 101	ICU stay—ND (2 RCTs, n = 101) MV—ND (2 RCTs, n = 101) ↓ Hospital stay (1 RCT, n = 41) ↑ MV (1 non-RCT, n = 60)	Adverse events—ND (2 RCT, n = 101) Mortality ND (2 RCT, n = 101) Need for inotropes ND (1 RCT, n = 41)
Human milk fortifier vs placebo [17]	1 RCT, n = 58	↑ Albumins and Prealbumins ↑ STRONG Kids score Hemoglobin ND	Preoperative adverse events—ND No NEC No death
Preoperative trophic breast milk feeding vs no enteral feeding [18]	1 RCT, n = 27	Postoperative feeding intolerance ND Nasogastric tube dependence	Postoperative NEC—ND GER medication—ND Mortality—ND
Preoperative nutrition support 2 weeks vs 1 week [16]	1 RCT, n = 40	↓ ICU stay ↓ Hospital stay ↓ MV ↑ Postoperative feeding intake other feeding related outcomes—ND ↑ Weight z-score pre- and postoperatively Height z-score—inconsistent Albumins—ND Hemoglobulin—ND	Sepsis—ND Successful extubation—ND Feeding-related adverse events ND
1-month preoperative nutrition support vs no support [19]	1 RCT, n = 80	↓ ICU and hospital stay ↓ Preoperative STRONG Kids Score ↑ Albumins, Prealbumins and Hemoglobulin	Not reported
Any preoperative feeding vs no feeding [26,27,31,32,33,34,35]	7 studies, n = 917	ICU stay—ND (3 studies, n = 226) ↓ Hospital stay (4 studies, n = 278) ↓ Days to achieve full feeds postoperatively (3 studies, n = 138) MV duration—inconsistent results (2 studies, n = 175) Postoperative weight z-score—ND (2 studies, n = 181) ↑ Albumins (1 study, n = 45) ↑ Children on full feeding (1 study, n = 235)	↓ CS-AKI (1 study, n = 347) NEC—ND (3 studies, n = 232), Postoperative infection rate—ND (1 study, n = 45) Mortality risk—ND (2 studies, n = 97)
Achieved vs not achieved center-recommended pre-operative caloric intake requirements [29]	1 study, n = 160	↑ Postoperative hospital stay	Not reported
Preoperative fortified nutrition (≥22 kcal/oz) vs unfortified nutrition (20 kcal/oz) [30]	1 study, n = 84	↑ ICU stay ↑ Hospital stay ↓ Postoperative weight MV duration—ND BMI-for-age percentiles at 5 and 10 years	Surgical site infection risk—ND

DHA, docosahexaenoic acid; CS-AKI, cardiac surgery-associated acute kidney injury; GER, gastroesophageal reflux; ICU, intensive care unit; IV, intravenous; LCT, long-chain triglycerides; MCT, medium-chain triglycerides; MV, mechanical ventilation; n, number of participants; ND, no difference; NEC, necrotizing enterocolitis; STRONG, Screening Tool for Risk on Nutritional status and Growth (pediatric nutritional risk screening tool); and RCT, randomized controlled trial.

## Data Availability

The original contributions presented in this study are included in the article/Supplementary Material. Further inquiries can be directed to the corresponding author.

## Supplementary Materials

The following supporting information can be downloaded at: https://www.mdpi.com/article/10.3390/nu18030544/s1, Figure S1: Effect of high-dose vitamin D supplementation vs. usual care/no intervention on total length of ICU stay (in days), Figure S2: Effect of high-dose vitamin D supplementation vs. usual care/no intervention on length of mechanical ventilation (in hours), Figure S3: Association between mean ICU length of stay in children receiving preoperative feeding compared to those without any preoperative feeding, Figure S4: Association between proportion of children fed preoperatively and shorter (<7 days) and longer (<14 days) stay groups, Figure S5: Association between any preoperative feeding and mortality risk. Table S1: Secondary outcomes, Table S2. Excluded studies with reasons, Table S3. Characteristics of included interventional trials, Table S4. Characteristics of included cohort and case-control studies, Table S5. Assessment of risk of bias in cohort studies assessed using Newcastle–Ottawa Quality Assessment Scale, Table S6. Assessment of risk of bias in case-control studies using Newcastle–Ottawa Quality Assessment Scale, Table S7. Summary of findings from study by Elegeresma et al., Table S8. Summary of findings using the GRADE approach. Reference [43] is cited in the Supplementary Materials.

## Abbreviations

The following abbreviations are used in this manuscript:

BDG	Bidirectional Glenn
BMI	Body mass index
CHD	Congenital heart disease
CI	Confidence interval
CS-AKI	Cardiac surgery-associated acute kidney injury
DHA	Docosahexaenoic acid
ERAS	Enhanced recovery after surgery
GER	Gastroesophageal reflux
GRADE	Grading of Recommendations, Assessment, Development, and Evaluation
HMF	Human milk fortifier
HR	Hazard ratio
ICU	Intensive care unit
ITT	Intention-to-treat
IV	Intravenous
LCT	Long-chain triglycerides
MD	Mean difference
MCT	Medium-chain triglycerides
MV	Mechanical ventilation
n	Number of participants
ND	No difference
NEC	Necrotizing enterocolitis
NeoC3	Neonatal Cardiac Care Collaborative
NOS	Newcastle–Ottawa Scale
OR	Odds ratio
PP	Per-protocol
PRISMA	Preferred Reporting Items for Systematic Reviews and Meta-Analyses
RCT	Randomized controlled trial
RoB 2	Revised Cochrane risk-of-bias tool for randomized trials
RR	Risk ratio
SD	Standard deviation
STRONG	Screening Tool for Risk on Nutritional Status and Growth
