# Supplementary material for: Preoperative Nutrition-Based Interventions in Children Undergoing Cardiac Surgeries—A Systematic Review and Meta-Analysis
Source: Nutrients. 2026 Feb 6;18(3):544. doi: 10.3390/nu18030544 (PMC12899530; doi:10.3390/nu18030544)
Supplement: Supplementary file 1 [file nutrients-18-00544-s001.zip › 10. Suppl Table S8. GRADE_05 Dec 2025.pdf]

Supplementary Table S8. Summary of findings using the GRADE approach

Certainty assessment							№ of patients		Effect		Certainty	Importance
**№ of studies**	**Study design**	**Risk of bias**	**Inconsistency**	**Indirectness**	**Imprecision**	**Other considerations**	**Intervention/Exposure**	**Comparator**	**Relative (95% CI)**	**Absolute (95% CI)**		
**NEC**												
3	non-randomised studies	serious[a]	not serious	not serious	serious[b]	none	14/113 (12.4%)	25/120 (20.8%)	**OR 0.57** (0.26 to 1.25)	**78 fewer per 1000** (from 144 fewer to 39 more)	⨁◯◯◯ Very low[a,b]	
**Length of ICU stay**												
2	non-randomised studies	serious[a]	not serious	not serious	very serious[b]	none	110	65	-	MD **3.56 lower** (7.22 lower to 0.11 higher)	⨁◯◯◯ Very low[a,b]	
**Length of hospital stay**												
4	non-randomised studies	very serious[c]	serious[d]	not serious	serious[e]	none	144	134	-	MD **-7.23 lower** (-14.07 lower to -0.4 lower)	⨁◯◯◯ Very low[c,d,e]	
**Mortality**												
2	non-randomised studies	serious[a]	not serious	not serious	very serious[b]	none	11/53 (20.8%)	13/44 (29.5%)	**OR 0.65** (0.25 to 1.69)	**81 fewer per 1000** (from 201 fewer to 119 more)	⨁◯◯◯ Very low[a,b]	
**Mean number of days to achiveve full feeds postoperatively**												

Supplementary Table S8. Summary of findings using the GRADE approach

Certainty assessment							№ of patients		Effect		Certainty	Importance
№ of studies	Study design	Risk of bias	Inconsistency	Indirectness	Imprecision	Other considerations	Intervention/Exposure	Comparator	Relative (95% CI)	Absolute (95% CI)		
3	non-randomised studies	very serious[c]	not serious	not serious	serious[f]	none	63	75	-	MD **3.29 lower** (4.23 lower to 2.34 lower)	⊕◯◯◯ Very low[c,f]	
**ICU LOS**												
2	randomised trials	not serious[g]	very serious[h]	serious[i]	serious[b]	none	51	50	-	MD **2.25 lower** (5.83 lower to 1.32 higher)	⊕◯◯◯ Very low[b,g,h,i]	
**Mechanical Ventilation**												
2	randomised trials	not serious[g]	very serious[h]	serious[i]	serious[e]	none	51	50	-	MD **22.02 lower** (58.86 lower to 14.82 higher)	⊕◯◯◯ Very low[e,g,h,i]	

CI: confidence interval; **MD:** mean difference; **OR:** odds ratio

Explanations

a. downgraded one level for study limitations: one of the three studies had moderate quality in NOS

b. downgraded two levels for substantial imprecision: wide confidence of intervals overlapping no effect, and the small sample size

c. downgraded two levels for study limitations: two studies had low or moderate quality in NOS

d. downgraded one level for substantial inconsistency: moderate heterogeneity, and minimal or no overlap of confidence intervals between some studies

e. downgraded one level for imprecision: small sample size, wide confidence intervals

f. downgraded one level for imprecision: small sample size

g. downgraded one level for study limitations: 1 of 2 trials had an unclear risk of bias in two domains (assessed with ROB-2)

h. downgraded two levels for substantial inconsistency: I2 may be considerable, and minimal or no overlap of confidence intervals was observed

i. downgraded for indirectness: differences in interventions (applicability), different dose administration
