# Supplementary material for: Preoperative Nutrition-Based Interventions in Children Undergoing Cardiac Surgeries—A Systematic Review and Meta-Analysis
Source: Nutrients. 2026 Feb 6;18(3):544. doi: 10.3390/nu18030544 (PMC12899530; doi:10.3390/nu18030544)

Supplementary Figure S1. Effect of high-dose vitamin D supplementation vs usual care/no intervention on total length of ICU stay (in days)

	Experimental			Control				Mean Difference
Study or Subgroup	Mean	SD	Total	Mean	SD	Total	Weight	IV, Random, 95% CI
McNally 2020	3.5	1.5	21	7.62	2.35	20	48.8%	−4.12 [−5.33, −2.91]
Sahu 2020	2.25	1.09	30	2.72	0.82	30	51.2%	−0.47 [−0.96, 0.02]
Total (95% CI)			51			50	100.0%	−2.25 [−5.83, 1.32]

Heterogeneity: $Tau^2 = 6.44$; $Chi^2 = 29.92$, df = 1 ($P < 0.00001$); $I^2 = 97\%$
Test for overall effect: Z = 1.23 (P = 0.22)

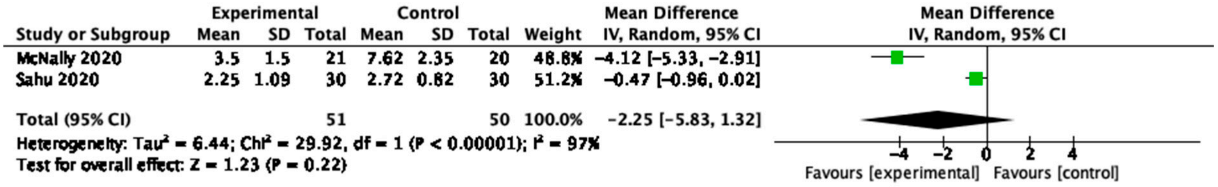

Supplementary Figure S2. Effect of high-dose vitamin D supplementation vs usual care/no intervention on length of mechanical ventilation (in hours)

	Experimental			Control				Mean Difference
Study or Subgroup	Mean	SD	Total	Mean	SD	Total	Weight	IV, Random, 95% CI
McNally 2020	19	11.5	21	61	44	20	47.0%	−42.00 [−61.90, −22.10]
Sahu 2020	22.4	15.81	30	26.74	15.79	30	53.0%	−4.34 [−12.34, 3.66]
Total (95% CI)			51			50	100.0%	−22.02 [−58.86, 14.82]

Heterogeneity: $Tau^2 = 649.27$; $Chi^2 = 11.84$, df = 1 ($P = 0.0006$); $I^2 = 92\%$
Test for overall effect: Z = 1.17 (P = 0.24)

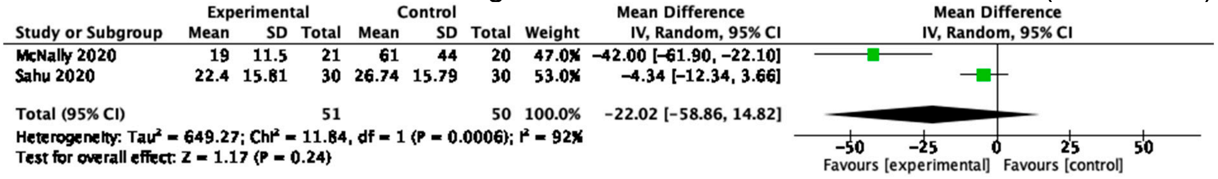

Supplementary Figure S3. Association between mean ICU length of stay in children receiving preoperative feeding compared to those without any preoperative feeding

	Experimental			Control				Mean Difference
Study or Subgroup	Mean	SD	Total	Mean	SD	Total	Weight	IV, Random, 95% CI
Scahill 2018	16	21	79	15	28	51	14.4%	1.00 [−7.97, 9.97]
Toms 2015	9.5	2.125	31	13.825	2.975	14	85.6%	−4.32 [−6.05, −2.60]
Total (95% CI)			110			65	100.0%	−3.56 [−7.22, 0.11]

Heterogeneity: $Tau^2 = 3.31$; $Chi^2 = 1.30$, df = 1 ($P = 0.25$); $I^2 = 23\%$
Test for overall effect: Z = 1.90 (P = 0.06)

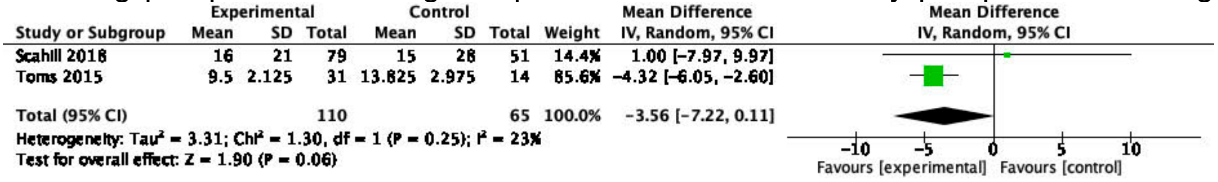

Supplementary Figure S4. Association between proportion of children fed preoperatively and shorter (<7 days), and longer (<14 days) stay groups

	Exposed to pre-op		Non-exposed to pre-op		Odds Ratio
Study or Subgroup	Events	Total	Events	Total	M-H, Random, 95% CI
Iliopoulos 2016	20	25	10	32	8.80 [2.57, 30.18]

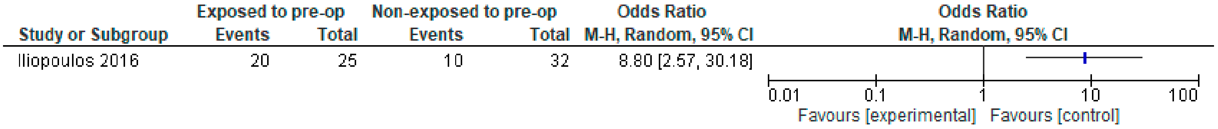

Supplementary Figure S5. Association between any preoperative feeding and mortality risk

	Exposed		Non-exposed			Odds Ratio
Study or Subgroup	Events	Total	Events	Total	Weight	M-H, Random, 95% CI
Toms 2015	6	31	4	14	42.9%	0.60 [0.14, 2.59]
Venna 2022	5	22	9	30	57.1%	0.69 [0.19, 2.44]
**Total (95% CI)**		**53**		**44**	**100.0%**	**0.65 [0.25, 1.69]**
Total events	11		13			

Heterogeneity: $Tau^2 = 0.00$; $Chi^2 = 0.02$, df = 1 ($P = 0.89$); $I^2 = 0\%$
Test for overall effect: Z = 0.89 (P = 0.37)

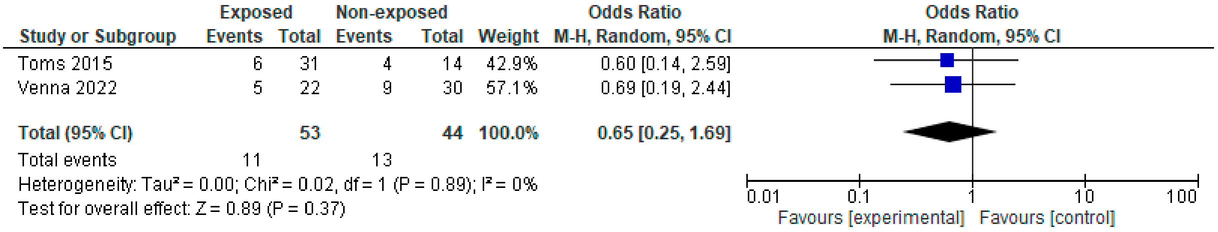

Supplement: Supplementary file 1 [file nutrients-18-00544-s001.zip › 11. Supplementary Figures_20 Dec.pdf]
