# Supplementary material for: Preoperative Nutrition-Based Interventions in Children Undergoing Cardiac Surgeries—A Systematic Review and Meta-Analysis
Source: Nutrients. 2026 Feb 6;18(3):544. doi: 10.3390/nu18030544 (PMC12899530; doi:10.3390/nu18030544)
Supplement: Supplementary file 1 [file nutrients-18-00544-s001.zip › 3. Suppl Table S1. Secondary outcomes.pdf]

Supplementary Table S1. Secondary outcomes

Assessment period	Outcomes
▪ **Preoperative**	▪ change in degree of malnutrition, measured with any of the following:<br>– anthropometry, reported as: weight-for-age, weight-for-height, height-for-age, BMI-for-age, head circumference-for-age z-scores or percentiles;<br>– any validated malnutrition risk score (e.g., STRONGkids score);<br>– - any laboratory markers of malnutrition (e.g., albumin, prealbumin concentration);<br>▪ compliance, measured as a percentage of compliant participants, during the intervention or as an adherence to exposure
▪ **Intraoperative**	▪ proportion of children with successful extubation;<br>▪ proportion of children with early extubation (<48h);<br>▪ proportion of children who required reintubation;<br>▪ total operating time;<br>▪ extracorporeal circulation time;<br>▪ proportion of children who required administration of inotropes drugs;<br>▪ proportion of children who required fluid therapy and its duration.
▪ **Postoperative**	▪ total length of hospital stay with any measures of assessment;<br>▪ change in degree of malnutrition (as described above);<br>▪ need and duration of mechanical ventilation;<br>▪ health-related quality of life, measured with any validated scale ;<br>▪ postoperative feeding tolerance (i.e., time to start, feeding route, frequency and volume), as reported by authors;<br>▪ postoperative complications (i.e., pulmonary, cardiovascular, infectious) as reported by authors;<br>▪ readmission rate;<br>▪ proportion of children who required enteral and parental feeding and its duration.
▪ **Any time during the follow-up period**	▪ adverse events related to intervention/exposure;<br>▪ mortality, as reported by authors (postoperative vs. pre- and intraoperative mortality was planned to be assessed separately, if feasible).
