# Supplementary material for: Preoperative Nutrition-Based Interventions in Children Undergoing Cardiac Surgeries—A Systematic Review and Meta-Analysis
Source: Nutrients. 2026 Feb 6;18(3):544. doi: 10.3390/nu18030544 (PMC12899530; doi:10.3390/nu18030544)
Supplement: Supplementary file 1 [file nutrients-18-00544-s001.zip › 4. Suppl Table S2. Excluded Studies with Reasons_08 Oct_AS.pdf]

Supplementary Table S2. Excluded studies with reasons

**Author year**	**Reason of exclusion**
Identification of studies via databases and registers	
Bassi 2024	Conference abstract
Bertrandt 2023	Conference abstract
Bhatia 2019	Conference abstract
Bhatia 2020	Conference abstract
Brown 2013	Conference abstract
ChiCTR2000031872	Study registry: not yet recruited
Day 2017	Conference abstract
Elgeresma 2024	Conference abstract, a final publication (Elgeresma 2023) was included
Fandinga 2023	Conference abstract
Garrido Martin 2012	Wrong population: adults
Hehir 2010	Conference abstract
Huh 2024	Wrong population: children with non-cardiac surgery
Jones 2022	Conference abstract
Kaplina 2023	Wrong exposure: lack of pre-operative nutrition exposure
Kataria-Hale 2019	Conference abstract
Killen 2019	Conference abstract
Killen 2018	Conference abstract
Larsen 2015	No outcome of interest
Makarenko 2016	Conference abstract
Marques 2023	Conference abstract
McNally 2018	Conference abstract, a final publication (McNally 2020) was included
Murray 2024	Conference abstract, a final publication (Murray 2025) was included
NCT05457712	Study registry: not yet recruited
Penk 2024	Conference abstract
Pentony 2023	Conference abstract
Rattanapittayaporn 2023	Wrong intervention: prolonged and normal fasting was not intervention of interest
Ruan 2024	Wrong exposure: feeding pattern assessed not in pre-operative period
Santos 2017	Wrong study design: no control group
Sahu2 023	Wrong population: only 30% of participants underwent cardiac surgeries
Singhal 2013	Conference abstract
Westin 2020	Wrong study design: a case-series
Van Niekerk 2023	Conference abstract

Supplementary Table S2. Excluded studies with reasons

Vergales 2020	Wrong exposure: pre-operative nutrition not assessed
Zhang 2022	Wrong intervention: only post-operative intervention
Identification of studies via other methods	
Becker 2015	Wrong exposure: enteral feeding only
Iannucci 2013	Wrong exposure: post-operative nutrition only
Kataria-Hale 2021	Wrong study design: lack of control group
Luce 2011	Wrong study design: no control group, enteral feeding assessed only as prognostic factor
Natarajan 2010	Wrong study design: case-series
Penk 2024	Wrong study design: a case-series (lack of control group)
Willis 2008	Wrong study design: case-series
Young 2025	Wrong study design: lack of control group (a historical control group only)
Yu 2021	Wrong exposure: only post-operative nutrition
