# Supplementary material for: Preoperative Nutrition-Based Interventions in Children Undergoing Cardiac Surgeries—A Systematic Review and Meta-Analysis
Source: Nutrients. 2026 Feb 6;18(3):544. doi: 10.3390/nu18030544 (PMC12899530; doi:10.3390/nu18030544)
Supplement: Supplementary file 1 [file nutrients-18-00544-s001.zip › 5. Suppl Table S3. Characteristics of interventional trials.pdf]

Supplementary Table S3. Characteristics of included interventional trials

Author, year, country	Study design of RCT (masking, allocation study setting)	Duration of study (follow-up),	Participants (age, preoperative type of cardiac diagnosis, type of surgical intervention, n)	Type of intervention (characteristics, dosage duration, frequency and type of administration, concomitant treatment, n)	Type of comparison (characteristics, n)	Type of outcomes (with time points reported)	Funding, sponsorship<br><br>Conflict of interest
**Trials assessing administration of various fatty acids emulsions**							
Bernabe-Garcia 2016, Mexico (1)	Single-center, double-blind, with two parallel arms.<br><br>NICU of Pediatric Hospital, Mexico City	Intervention/control: 2 days before surgery, suspended 4 h before surgery, resumed and continued for 6 days after surgery.<br><br>Follow-up: until NICU discharge.	Newborns aged below one month, with cardiovascular malformations; scheduled for Blalock–Taussig shunt or aortoplasty; >34 weeks gestational age; adequate weight; tolerating enteral feeding.<br><br>n/N=34/55<br><br>Median age at enrollment: 18.8±13.7 days for DHA; and 21.3±10.1 days for sunflower oil group	DHA (75 mg/kg of baseline weight/day), in sunflower oil (Neuromins for Kids, Marek Life Enriched Inc., Parsipany, N.J., USA); administered in 2 daily doses, via enteral feeding tube – with a syringe before human milk or formula feeding.<br><br>n/N=16/27.	Sunflower oil, with similar dosage, consistency, appearance and method of administration<br><br>n/N=18/28.	Primary outcome: development of severe sepsis and cardiovascular and respiratory organ dysfunctions (ODs).<br>Secondary outcomes: hematologic, hepatic and renal ODs, length of NICU stay.<br><br>Others: biological markers (percentage of positive mononuclear cells for IL-1β, TNF-α, IL-6, IL-10, IL-1ra; CD69 on CD4+ lymphocytes), severity of illness, duration of cardiovascular surgery, aortic clamp, concurrent medications, total parenteral nutrition, human milk and formula intake; adverse events.	Funded by grants from FIS/IMSS/PROT 094 and CONACYT Salud-2011-1-161643 (Mexico).<br><br>COI: Nothing to disclose.
Larsen 2012, Canada (2)	Double-blind with two parallel arms;<br><br>Pediatric neonatal and paediatric ICU	1-4 days before surgery and 10 days post-operatively	Infants with CHD undergoing open-heart surgery with cardiopulmonary bypass; appropriate for gestational age, Apgar score >7 at 5 and 10 min, no infection pre-operatively<br><br>n/N=32/36 (four excluded after randomization, did not receive intervention)<br><br>Mean age: 10.6 days (range 3-66)	An emulsion containing 50% MCTs and 40% LCTs from soybean oil and 10% of fish oil (0.02 g of EPA/DHA per ml of total fat; MLF 541, Lipoplus; B. Braun Melsungen, Melsungen, Germany); intravenous administration<br><br>Initiated at 0.5 g/kg/d and gradually increasing to a max. of 3.5 g/kg/d, along with amino acid/dextrose solutions.<br><br>n=16 (analyzed)	Emulsion composed exclusively of LCTs from soybean oil lipid (Intralipid®; Clintec Nutrition Company, Baxter, Deerfield, IL);<br><br>n=16 (analyzed)	Primary outcome: difference in TNF-alfa and IL-6.<br><br>Secondary outcomes (clinical outcomes): Ventilator Days, PICU Days, Hospital Days, Days to sternal closure, steroids post-operatively, antibiotic days, PRISM III score, inotrope score on Day 1 post-operatively, sepsis, culture – positive, culture – negative	Funded by the Stollery Children's Hospital Foundation, the Canadian Institute of Health Research and Natural Sciences and Engineering Research Council of Canada. B Braun Melgusen, Germany supplied Lipoplus 5:4:1.<br><br>One author received payment for manuscript preparation, others had nothing to declare.
**Trials assessing administration of high-dose cholecalciferol**							

Supplementary Table S3. Characteristics of included interventional trials

Randomized controlled clinical trial							
McNally 2020, Canada (3)	Double-blind, with two parallel arms, pilot and feasibility trial;<br><br>Canadian tertiary center	From the enrollment day until discharge (intervention – from enrollment to the day of discharge)	Children aged 36 weeks corrected GA to 17 years with CHD who required cardiopulmonary bypass within the next 12 months<br><br>n/N=41/46<br><br>Median age: 4.6 (1.0–45.6) months in intervention group and 3.8 (0.8–35.6) months in control group	Cholecalciferol (vitamin D3) enteral high dose (based on the IoM tolerable upper intake level, eqv. to 1600 IU <1 y or 2400 IU for aged 1 to 17 y per day)<br>n/N=21/24	Usual care arm: the adequate intake dose for infants or recommended daily allowance for children >1 y. (< 1 year = 400 IU/day, > 1 year = 600 IU/day)<br><br>Formula-fed infants received placebo (0 IU/day) in addition to formula intake (~400 IU).<br><br>n/N=20/22	Primary feasibility outcomes: proportion of participants with an immediate post-operative serum 25OHD concentration under 50 nmol/L.<br><br>Additional outcomes: vitamin D related adverse events, including: hypercalcuria, hypercalcemia and nephrocalcinosis; accrual date, withdrawal rate, non-adherence to the study protocol, ability to maintain allocation and blinding; post-operative clinical outcomes included mortality,<br>fluid intake, hypocalcemia, catecholamine infusion requirements, arrhythmias, positive bacteria culture,<br>renal failure, and duration of mechanical ventilation,<br>pediatric ICU, and hospital stay; the Pediatric Risk of<br>Mortality (PRISM).	Funded by a Grant-in-Aid from the Heart and Stroke Foundation of Canada and a research award from the Children's Hospital of Eastern Ontario Research Institute.<br><br>COI: One author was employed by Euro-Pharm International Canada Inc., which developed and prepared the concentrated vitamin D solution used in the study.
Sahu 2020, India (4)	With two parallel-arms; blinding not reported<br><br>Department of Cardiothoracic and Vascular Surgery, AIIMS, New Delhi	Intervention: 2 weeks ± 1 wk after vit D administration; in control group operated at the same time sitting depending on waiting list and date allocation by the surgeon<br><br>Follow up: until discharge from ICU	Children aged between 6 months and 18 years with CHD TO, who underwent intracardiac repair with CPB;<br><br>n=60<br><br>Mean age: 5.96 ± 2.88 years in intervention group and 6.52 ± 3.55 years in control group	Oral cholecalciferol (vitamin D3 as granular powder form, Troika Pharmaceuticals Ltd., Ahmedabad, Gujarat, Indie), administered as a "stoss therapy" – a single megadose at 10,000 IU/kg bw up to 400,000 IU. orally in milk<br><br>n=30	No vitamin D supplementation<br><br>n=30	Primary outcome: immediate postoperative serum vit D levels.<br><br>Secondary outcomes: postoperative duration of inotrope requirement, mechanical ventilation, ICU stay, infection rate, and vit D-related adverse effects: including: hypercalcemia and hypercalciuria.	Funded from the institute intramural fund.<br><br>COI: Nothing declared.
Non-randomized controlled clinical trial							
Jelveh-Moghaddam 2020, Iran (5)	Two parallel arms; blinding not reported<br><br>Tertiary hospital (Shahid Modarres	Intervention: 3-day preoperatively<br><br>Follow up- until discharge	Children under 16 years of age, with confirmed CHD undergoing cardiac surgery with deficient level of vitamin D	Group B (with vit D deficiency): vit D treatment (a one-time treatment of 300,000 IU IM) 3 days before surgery up to normal levels (> 30 ml) before surgery	Group A (with vit D deficiency): no treatment<br>n=30 (analyzed)	ICU/hospital stay (assessed but not reported), mechanical ventilation time, VIS score, mortality<br><br>Other outcomes, but out of scope of this review: Cytokines (IL-1, IL-6, IL-10, TNF-α) pre-op, 6h and 24h post-CPB; hemodynamics- systolic	Supported by Anesthesiology Research Center, Shahid Beheshti University, Iran<br><br>No COI

Supplementary Table S3. Characteristics of included interventional trials

	Hospital, Tehran)		Analyzed=90 (30 per group)<br><br>Patients with insufficient vit D3 level received intramuscular 300.000 IU of vit D3 before the surgery and those who achieved ≥30 ng/ml were included and divided into 3 groups.<br><br>Mean age in group A and B: 3.97±2.2 and 2.79±1.9 years respectively	n=30 (analyzed)<br><br>Group C: normal baseline vit. D, no treatment (n=30)		and diastolic blood pressure, heart rate, SpO2, ECG; metabolic measurements- hematocrit level, lactate level, PaCO2, minute ventilation time, and intravenous fluid therapy	
**Trial assessing administration of human milk fortifier (HMF)**							
Lin 2022, China (6)	Double-blind, with two parallel arms.<br><br>Provincial hospital in China	1-month after the intervention, before the cardiac surgery.	Infants with non-restricted VSD (diagnosed in a study hospital after birth and were followed up in the outpatient clinic 1 mo later), with no congenital malformation associated with essential organs. All patients were breastfed. All patients underwent surgical repair of VSDs after the study ended.<br><br>n=58 infants (29 per group)	Human milk fortifier (HMF, Nestle, Germany) added to expressed breastmilk in gradually increasing doses: ¼ (25 ml human milk: 0.25 g HMF), after good tolerance confirmed for 3 days, dose was increased - ½, then full (1g HMF/25 ml human milk), after that the HMF was added at this fixed-rate and infants were fed on demand<br><br>n/N=29/29	Placebo (maltodextrin) added to expressed breastmilk; feeding method the same as that in the intervention group<br><br>n/N=29/29	The nutritional status (weight, head circumference, height, albumin, prealbumin, hemoglobin, STRONGkids score) assessed at 1 month and complications between the two groups after the intervention. Preoperative complications (pneumonia, liver insufficiency, feeding intolerance, jaundice, necrotizing enterocolitis, gastrointestinal bleeding, death).	Funded by promotion of appropriate technology projects to Rural and Urban Communities, Fujian Province China (2020TG007)<br><br>COI: not reported
**Trial assessing administration of any preoperative nutrition-based support**							
El-Ganzoury 2020, Egypt (7)	Open-label, with two parallel arms.<br><br>Pediatric cardiac surgery ICU at Ain Shams University Hospitals.	2-wk or 1-wk preoperatively until discharge	Infants with CHD admitted to the cardiothoracic unit for either palliative or corrective surgery and with nutritional deficiencies with moderate or severe malnutrition (WAZ ≥ -2)	2-week nutrition prehabilitation;<br>- unweaned infants: ONS (Infatrini, Nutricia, Erlangen, Germany) added to expressed breast milk as overnight feed / in formula-fed: 1m;/kg hourly for the first 4h to	1-week nutrition prehabilitation (the same as intervention)<br>n=20<br><br>For both groups: Supplemental PN was added to reach the target	Primary outcome data included daily feeding characteristics, anthropometric, and laboratory measures.<br><br>Secondary outcomes included postoperative vital stability, surgical complications, nosocomial sepsis, inotropes' withdrawal, duration (h). of mechanical ventilation (MV) and	Funding: not reported<br><br>COI: nothing declared.

Supplementary Table S3. Characteristics of included interventional trials

			n=40<br><br>Median age: 1.25 (0.93.34) years in intervention group and<br>0.8 (0.551.25) years in control group	be increased gradually by 1 ml/kg every 4h to reach the max hourly amount of 6 ml/kg if tolerates; if target calorie intake could not be reached, high-calorie dense formula (Infantrini) was used.<br>≥6 mo of age: high calorie-dense formula (Infantrini for children 6-12 mo, Pediasure, Abbott Laboratories, Chicago, IL, USA and Nutrini, Nutricia for children ≥1 y) or ordinary feeding with supplemental feeds with a high calorie density formula.<br><br>n=20	fluid and energy requirement if not achieved by EN.<br>Both groups continued nutrition support postoperatively after discharge.	extubation timing, ICU length of stay (LOS; h), and total hospital LOS (d).	
Xu 2022, China (8)	Open-label, only outcome assessors were blinded;<br><br>Two tertiary hospitals in China	1-month preoperative nutritional support; and post-operatively follow-up to discharge	Infants with non-restrictive ventricular septal defect; condition relatively stable and the operative treatment was performed within 1-2 months<br>n/N=80/80<br><br>Mean age: 36.7±8.6 days in intervention group and 35.2±8.8 days in control group	Nutritional support 1-month before the expected time of surgery<br><br>After admission all infants were bottle-fed with 120-150 kcal/kg/day (targeting feeding dose), if target dose could not be reached with breast milk or formula, HMF or high-calorie milk powder was added, respectively. In child with feeding difficulties, NG tube feeding.<br><br>If target calorie could not be achieved by EN, PN was added:<br><br>n/N=40/40	No nutritional support plan, the feeding process and feeding method were led by their parents; fed on demand without PN support.<br><br>n/N=40/40	Primary outcomes: body weight, head circumference, STRONGkids score, albumin, prealbumin, hemoglobin at admission and one month after the intervention.	Not reported.
**Trial assessing administration of preoperative trophic breast milk feeding**							

Supplementary Table S3. Characteristics of included interventional trials

Zyblewski 2015, USA (9)	Pilot study, single-blinded (personnel); PCICU/NICU at Medical University of South Carolina	Pre-operatively until the day (until midnight before surgery) of hospital discharge	Term-born neonates (≥37 wks gestation) with structural heart disease who required CPB surgery before hospital discharge<br><br>n/N=27/27<br><br>intervention group age, mean (range): 6 (4-9) days<br><br>control group age, mean (range): 7 (4-8) days	Pre-operative feeding trophic breast milk feeds every 3h, for a total daily volume of 10 ml/kg/day via NG; the feeding was discontinued at midnight before the scheduled surgery.<br><br>Both groups received PN pre- and postoperatively in accordance with standard PCICU CPG until full enteral feed was achieved.<br><br>n/N=14/14	No enteral feeding preoperatively<br><br>n/N=13/13	Intestinal permeability measured by urinary lactulose/mannitol (L/M) ratio at 3 timepoints (pre-op, POD7, POD14); intraoperative course, nutrition delivery, postoperative enteral feeding data (weight at discharge, formula change for feeding intolerance, NG tube dependence at discharge, exclusive breast milk feeds at discharge), Gi complications (gastroesophageal reflux medication at discharge); adverse events, including mortality, NEC	Supported by National Center for Advancing Translational Sciences (UL1TR000062)<br><br>COI: nothing declared.
AIIMS, All India Institute of Medical Sciences; CHD, congenital heart disease; COI, conflict of interest; CPB, cardiopulmonary bypass; CPG, clinical practice guideline; CS-AKI, cardiac surgery–associated acute kidney injury; DHA, docosahexaenoic acid; ECG, electrocardiogram; EN, enteral nutrition; EPA, eicosapentaenoic acid; GA, gestational age; GER, gastroesophageal reflux; GI, gastrointestinal; HMF, human milk fortifier; ICU, intensive care unit; IL, interleukin; IL-1β, interleukin 1 beta; IL-1ra, interleukin-1 receptor antagonist; IL-6, interleukin 6; IL-10, interleukin 10; IM, intramuscular; IoM, Institute of Medicine; IV, intravenous; L/M ratio, lactulose/mannitol ratio; LCT, long-chain triglycerides; LOS, length of stay; MCT, medium-chain triglycerides; MV, mechanical ventilation; n, number of participants; ND, no difference; NEC, necrotizing enterocolitis; NG, nasogastric; NICU, neonatal intensive care unit; OD(s), organ dysfunction(s); ONS, oral nutritional supplement(s); $PaCO_2$, partial pressure of carbon dioxide in arterial blood; PCICU, pediatric cardiac intensive care unit; PICU, pediatric intensive care unit; POD, postoperative day; PN, parenteral nutrition; PRISM (PRISM III), Pediatric Risk of Mortality (score); RCT, randomized controlled trial; SpO_2, peripheral oxygen saturation; STRONGkids, Screening Tool for Risk on Nutritional status and Growth; TNF-α, tumor necrosis factor alpha; VIS, vasoactive–inotropic score; VSD, ventricular septal defect; WAZ, weight-for-age z score; 25OHD, 25-hydroxyvitamin D.							

References:

1. Bernabe-Garcia M, Lopez-Alarcon M, Villegas-Silva R, Mancilla-Ramirez J, Rodriguez-Cruz M, Maldonado-Hernandez J, et al. Beneficial Effects of Enteral Docosahexaenoic Acid on the Markers of Inflammation and Clinical Outcomes of Neonates Undergoing Cardiovascular Surgery: an Intervention Study. Annals of nutrition & metabolism. 2016;69(1):15-23.
2. Larsen BM, Goonewardene LA, Joffe AR, Van Aerde JE, Field CJ, Olstad DL, et al. Pre-treatment with an intravenous lipid emulsion containing fish oil (eicosapentaenoic and docosahexaenoic acid) decreases inflammatory markers after open-heart surgery in infants: a randomized, controlled trial. Clinical nutrition (Edinburgh, Scotland). 2012;31(3):322-9.
3. McNally JD, O'Hearn K, Fergusson DA, Lougheed J, Doherty DR, Maharajh G, et al. Prevention of post-cardiac surgery vitamin D deficiency in children with congenital heart disease: a pilot feasibility dose evaluation randomized controlled trial. Pilot and Feasibility Studies. 2020;6(1).

Supplementary Table S3. Characteristics of included interventional trials

4. Sahu MK, Bipin C, Niraghatam HV, Karanjkar A, Singh SP, Rajashekar P, et al. Vitamin D Deficiency and Its Response to Supplementation as Stoss Therapy in Children with Cyanotic Congenital Heart Disease Undergoing Open Heart Surgery. Journal of Cardiac Critical Care. 2019;3(1):17-23.
5. Jelveh-Moghaddam H, Fani K, Hekmat M, Azari AA. The effects of Vitamin D3 in pediatric patients undergoing congenital heart surgery. Journal of cellular and molecular anesthesia. 2020;5(2):66-73.
6. Lin YF, Lin WH, Lin SH, Zhang QL, Chen Q, Zheng YR. Using Human Milk Fortifiers to Improve the Preoperative Nutritional Status of Infants With Non-restricted Ventricular Septal Defect. Frontiers in Pediatrics. 2022;10.
7. El-Ganzoury MM, El-Farrash RA, Ahmed GF, Hassan SI, Barakat NM. Perioperative nutritional prehabilitation in malnourished children with congenital heart disease: a randomized controlled trial. Nutrition (Burbank, Los Angeles County, Calif). 2021;84:111027.
8. Xu LP, Lin SH, Zhang QL, Zheng Y, Lin G. Short-Term Nutritional Support Improves The Preoperative Nutritional Status of Infants With Non-Restrictive Ventricular Septal Defect: A Prospective Controlled Study. Heart Surg Forum. 2022;25(5):E745-e9.
9. Zyblewski SC, Nietert PJ, Graham EM, Taylor SN, Atz AM, Wagner CL. Randomized Clinical Trial of Preoperative Feeding to Evaluate Intestinal Barrier Function in Neonates Requiring Cardiac Surgery. Journal of pediatrics. 2015;167(1):47-51.e1.
