# Supplementary material for: Preoperative Nutrition-Based Interventions in Children Undergoing Cardiac Surgeries—A Systematic Review and Meta-Analysis
Source: Nutrients. 2026 Feb 6;18(3):544. doi: 10.3390/nu18030544 (PMC12899530; doi:10.3390/nu18030544)
Supplement: Supplementary file 1 [file nutrients-18-00544-s001.zip › 6. Suppl Table S4. Characteristics of observational studies.pdf]

Supplementary Table S4. Characteristics of included cohort and case-control studies

**Author, year, country(-ies), *n***	**Study design, duration of study (follow-up), recruitment**	**Participants (age, preoperative type of cardiac diagnosis, type of surgical intervention, n)**	**Exposed participants, n**	**Non-exposed participants (controls), n**	**Confounders**	**Type of outcomes of interest (with time points reported, adjusted and non-adjusted)**	**Funding, sponsorship<br><br>Conflict of interest**
**Cohort studies**							
Bertrandt 2024 (Pediatric Cardiac Critical Care Consortium [PC4] and NEPHRON network; USA, 22 hospitals) (1)	A secondary (retrospective) analysis of multicenter cohort study (the NEPHRON), patients between 2015 and 2018	Neonates (aged <30 days) who underwent stage 1 palliation (S1P)<br><br>HLHS: 74%<br><br>N=347<br><br>Median age at surgery: 5 days (IQR 4-7)<br>Median weight: 3.2 kg (IQR 2.9-3.5)	Children who received pre-operative enteral feeding<br>n=130 (37%)<br><br>*In primary analysis:*<br>Neonates with CS-AKI (n=231, 67%; of which severe AKI stage 2–3: n=90), undergoing S1P	Children who did not receive pre-operative enteral feeding<br>n=217 (63%)	Fluid-corrected severe AKI (stage 2-3 vs 0-1), AKI score chromosomal anomalies, inotropes or mechanical ventilation at time of surgery, Vasoactive-Inotropic Score >15 on POD0, 1st day of negative fluid balance >POD1, prematurity, major postoperative complication or infection	Primary outcome: CS-AKI incidence (KDIGO, POD 0–6), severe CS-AKI.<br><br>Other outcomes: hospital mortality.	One author's institution received funding from Bioporto Diagnostics and the Gerber Foundation.<br><br>Other authors reported no COI.
Dabbagh 2024 (USA, single center) (2)	Retrospective single-center cohort study, data from congenital cardiac surgery database between Jul-2017 and Jun-2022<br><br>Follow-up: up to discharge	Infants ≤30 days who underwent a single cardiac surgery and survived to discharge<br><br>Varied diagnosis: hypoplastic aortic arch 28%, single-ventricle CHD 31%, conotruncal CHD 31%, other CHD 10%<br><br>N=235	Infants receiving any preoperative oral feeding (defined as any oral intake up to the day of cardiac surgery (n=178, 76%) — subdivided into trophic ≤20 ml/kg/day (n=91) and >20 ml/kg/day (n=87)	Infants not fed preoperatively (n=57, 24%)	Variables that were clinically relevant and significantly associated with full oral intake on discharge were included as variables in a multivariable logistic regression model (i.e., statistically significant demographics and peri-operative factors)	Primary: discharge feeding status (full oral vs. tube feeding: NG or GT).<br><br>Secondary: dose–response of preop oral intake	Authors reported no COI.
Elgersma 2023 (USA, 68 pediatric cardiology centers; National	Retrospective registry-based cohort study using a propensity score	Infants with SV CHD who completed Stage 1 palliation (S1P)	Human milk feeding (maternal/donor via any route) and direct breastfeeding (directly from a lactating person) Categorized as:	Infants with low duration/no HM or direct BF at the same time points (propensity-score matched controls)	Propensity score models included infant demographics (sex, race, GA, weight-for-age z-score), CHD/clinical status (preop instability, intubation, postop complications), socioeconomic factors (insurance type, income, social deprivation index, rural/urban), and site-level practices	Outcomes: Pre- and post-operative NEC, infection-related complications, sepsis, time to full feeds, interstage readmission	Supported by the National Institute of Health; no payment or services from third parties received by

Supplementary Table S4. Characteristics of included cohort and case-control studies

Pediatric Cardiology Quality Improvement Collaborative [NPC-QIC] registry) (3)	matched cohort analysis<br><br>Data from the NPC-QIC registry between 2016 and 2021<br><br>Follow-up: up to from pre-S1P time to 1 year of age	Primary diagnosis: HLHS 70.5%, other SV 29.5%<br><br>N=2491	exclusive HM (n=934)/any DBF (n=378) vs not exclusive HMF/no DBF<br>high HM/DBF duration versus low HM/DBF duration; high HM duration: exclusive preop HM + any HM at discharge (n=603), high DBF duration: any preop DBF + any DBF at discharge (n=102)<br><br>Mean age at S1P admission (SD): 1.13 year (4.87)	Not exclusive HM (n=331), no DBF (n=920); low HM duration: any type of preop feeding but no HM at discharge (n=464); low DBF duration: Any type of preop feeding but no DBF at discharge (n=1004)		for feeding-related complications, hospital length of stay (S1P and S2P hospitalization), all-cause mortality, number of unplanned interstage readmissions, interstage readmission for major adverse event	authors or institutions; one author reported to be: advisory board member and receiving speaker honorarium from Medela Americas, other authors no COI.
Menon 2013 (USA, National Pediatric Cardiology Quality Improvement Collaborative Registry, 31 hospitals) (4)	Multicenter registry-based retrospective cohort study; data from patients discharged between Dec 2009–Aug 2010<br><br>Follow-up trough BDG hospitalization	Infants with HLHS or complex single-ventricle, undergoing Norwood, /Norwood-variant, and underwent BDG discharged home<br><br>N=162<br><br>33% aortic and mitral atresia<br><br>Median age: 155 days (range 78-128)	Infants who met center-defined caloric intake recommendations before surgery (as determined by the individual center at the last hospital/clinic visit before BDG)	Infants who did not meet center-defined caloric intake recommendations before surgery	The pre-BDG variables considered as potential risk factors for adverse outcome<br><br>Birth weight, sex, ethnicity, race, cardiac anatomical diagnosis, type of Norwood palliation (Sano vs. modified Blalock–Taussig shunt), home surveillance (yes/no), met center-defined target recommended caloric intake at the most recent hospital discharge or clinic visit before BDG (yes/no), age at BDG, weight-for-age z-score at BDG, length-for-age Z-score at BDG, oxygen saturation at BDG, and type of BDG. Death or cardiac transplantation during the first 30 postoperative days or before discharge from BDG hospitalization	Outcomes: BDG complications; resource use: days of hospitalization, ventilation, inotrope use, postoperative complications	Supported by the Joint Council for Congenital Heart Disease NPC-QIC quality improvement collaborative<br><br>Industry funding and COI not reported
Murray 2025 (USA) (5)	Retrospective single-center cohort study, data from patients between Jan 2010–Dec 2013<br><br>Cohorts were analyzed at 0–30 and 31–60 days post-repair as	Infants aged <18 months at the time of surgery, with ASD, VSD, AVSD or TOF, undergoing surgical repair<br><br>N=84	Infants receiving fortified nutrition preoperatively ≥22 kcal/oz (n=24, 28.6%) at the time of surgical repair	Infants receiving unfortified nutrition (20 kcal/oz) (n=60, 71.4%) at the time of surgical repair	Subgroup analysis: patients with tetralogy of Fallot and patients with ASD, VSD, or AVSD.	Primary: growth velocity, change in weight-for-age percentile<br><br>Secondary outcomes: occurrence of surgical site infection, duration of intubation, length of CICU stay, length of hospital stay; BMI-for-age percentiles.	Authors reported no COI.

Supplementary Table S4. Characteristics of included cohort and case-control studies

	well as at 2, 5, and 10 years of age.	ASD, VSD, ASD +VSD: 61.9%<br>TOF, TOF + ASD: 29.8%<br>AVSD: 8.3%<br><br>Median age at repair: 4 (range: 3-6)					
Scahill 2017 (USA) (6)	Retrospective single-center cohort study (chart review), from patients between Jul 2011 and Jul 2013<br><br>Follow up- until discharge	Neonates aged ≤31 days, admitted to the pediatric CICU, undergoing cardiac surgery<br><br>N=130<br><br>19% (24/130) HLHS; 42% (54/130) other single ventricle physiology<br><br>Mean age at surgery: 8 days (13)	Neonates receiving preoperative enteral feeding (any amount; n=79, 61%):<br>- trophic feeds ≤20 cc/kg/day (n=33),<br>- >20 ml/kg/day (n=32),<br>- unknown volumes (n=14)	No pre-operative enteral feeding (n=51, 39%)	Single ventricle physiology, structural gastrointestinal abnormality, confirmed genetic abnormality, presence of umbilical artery catheter, need for inotropic support during the preoperative period, ductal dependence for systemic circulation and gestational age < 37 weeks	Primary: NEC (Modified Bell stage ≥IIa)<br><br>Association between volume of feeds and NEC<br><br>NEC incidence., growth failure, measured by change in weight for age Z score (WAZ) from hospital admission to hospital discharge, total ventilator dependent days, total hospital length of stay and total intensive care unit length of stay	Supported by the National Heart, Lung and Blood Institute of the Nationat Institute of Health.<br><br>Not represent view of a funder (NIH).<br><br>Reported lack of the COI. .
Toms 2015 (USA) (7)	Retrospective single-center cohort study (cardiac ICU database), patients between 2008 and 2012; consecutive neonates with HLHS<br><br>Follow up- from preoperative period (median day 2) to discharge	Neonates with hypoplastic left heart, undergoing Norwood palliation<br><br>Most common: mitral atresia, aortic atresia: 43%,<br><br>N=45	Preoperative bolus feeds – orally or via NG tube every 3 h (typically 10 ml for a total of 20–30 mL/kg/day, breast milk or CMP formula) ≥2 days; n=31 (69%), patient who received TFs consistently for longer than 2 days<br><br>Decision to initiate feeds was based on attending preference. The remaining nutrition consisted of total PN that was advanced daily to achieve a goal of 110	No preoperative feeds (NPO), n=14 (31%)<br><br>All patients had protocolized feeds postoperatively, with initiation via transpyloric tube.	Not reported.	Survival to discharge, duration of MV, peak inotrope score, postoperative infection, day of first postoperative feed, day of full feeds, ICU length od stay, hospital length of days<br><br>CPB time, minutes<br>ACC time, minutes<br>OR fluid balance, mL/kg<br>Peak lactate, mmol/L<br>Fluid balance at 48 h, mL/kg, low ALC first 72 h, lowest album in first 72 h	Financial support from departmental funds (University of Alabama at Birmingham); authors reported no COI.

Supplementary Table S4. Characteristics of included cohort and case-control studies

			mL/kg/d with 3 g/kg/d of protein and 3 g/kg/d of lipids.				
Venna 2022 (USA)(8)	Prospective single-center cohort study, patients between Aug 2017 and Dec 2020; implementation of standardized feeding protocol<br><br>From S1P through postoperative hospitalization<br><br>Episodes of NEC that occurred at any time point before or after S1P, but prior to death or S2P , were included.	Neonates with single ventricle lesions undergoing stage I ventricle palliative repair (including: Norwood, BlalockTaussig shunt, Hybrid, pulmonary artery band/bands, and PDA stent); aged ≥37 weeks<br><br>N=52/60<br><br>39% HLHS,<br>15% atrioventricular canal<br>46% other	Preoperatively fed infants according to established hospital feeding protocol n=22/26 (85%)<br><br>receiving human milk (27%; n=6), formula (41%; n=9), or both (32%, n=7)<br><br>.	Infants not preoperatively fed (n=30, 58%) due to clinician preference or clinical deterioration	Birth weight, gestational age, race and ethnicity groups, primary care diagnosis, surgery type	Preoperative and intraoperative clinical data included ventricular function, cardiac arrest, need for extracorporeal membrane oxygenation (ECMO), prostaglandin dependence, age at surgery and surgery type. Postoperative clinical events were reported and included length of hospital stay, time to goal feed, vocal cord paralysis, diaphragm paralysis, arrhythmia, cardiac arrest, ECMO, G-tube placement, NEC, and mortality. NEC was categorized by all stages I–III; time to full feed (time to reaching a goal volume of 100 ml/kg).	No specific funding reported<br><br>One author reported to be a consultant of Actelion Pharmaceuticals. The other authors declared that they have no COI.
Zacharias 2025 (USA)(9)	Retrospective single-center cohort study based on chart analysis of patients between Jan 2020 and Mar 2023<br><br>Follow up: to the date of discharge or transfer to Cardiothoracic ICU (CTICU) for surgical intervention.	infants with moderate/severe CHD (born with HLHS, Tetralogy of Fallot (ToF) with or without pulmonary AVSD, DORV, TGA, large PDA, VSD, coarctation of the aorta, or severe valvular disease. – following ICD-10), requiring surgery, ≥7 days NICU stay	Infants managed with standardized preoperative feeding protocol (n=12)<br><br>Protocol #1: mild abnormal cardiac function, achieving full feeds by day 7 of life.<br><br>Protocol #2 – moderate to severe abnormal cardiac function – initiation of feed less than trophic 20 ml/kg/d, achieving full feeds by day 12 of life.	Infants managed without standardized protocol (n=39)<br><br>'non-protocol' – born prior to the creation of the center's feeding protocol and with no protocol utilized despite the institutionalization of the protocol	Not reported.	Growth velocity at discharge, anthropometric z-score changes (WAZ, LAZ, HAZ – NS) at discharge since birth, NEC incidence, time to full enteral feeds, NEC incidence, NICU length of stay, DOL when regained birthweight	No external funding reported.<br><br>Authors reported no COI.

Supplementary Table S4. Characteristics of included cohort and case-control studies

		N=51<br><br>HLHS and critical stenosis or coarctation: 16.7% and 17.9% in protocol and non-protocol group						
**Case-control study**								
**Author, year, country(-ies), *n***	**Study design, duration of study (follow-up), recruitment**	**Participants (mean age, preoperative type of cardiac diagnosis, type of surgical intervention, number)**	**Exposed participants (Cases)**	**Non-exposed participants (controls)**	**Confounders**	**Exposure(s) of interest**	**Type of outcomes of interest**	**Funding, sponsorship**<br><br>**Conflict of interest**
Iliopoulos 2016 (USA)(10)	Single center case-control study, patients between Nov 2001 and Nov 2011 undergoing ASO for TGA<br><br>Follow up: to the date of discharge home.	Neonates undergoing ASO for TGA (with or without VSD or coarctation of aorta)<br><br>N=105<br><br>(78 ASO, 25 ASO with ventricular septal defect (VSD) closure and 4 ASO with VSD closure and aortic coarctation repair)	Neonates undergoing ASO for TGA with postoperative stay less than 7 days- short stay (SS) group<br><br>N=25	Neonates undergoing ASO for TGA with postoperative stay more than 14 days- long stay (LS) group<br><br>N=32	Preoperative intubation, preoperative PGE1 infusion, preoperative ballon atrial septostomy, preoperative inotropes infusion	Preoperative enteral feeding – 80% in SS group, 31% in LS group	Postoperative hospital length of stay	No external funding reported.<br><br>Authors reported no COI.

ACC, aortic cross-clamp; AKI, acute kidney injury; ALC, absolute lymphocyte count; ASD, atrial septal defect; ASO, arterial switch operation; AVSD, atrioventricular septal defect; BDG, bidirectional Glenn; BMI, body mass index; CHD, congenital heart disease; CICU, cardiac intensive care unit; CS-AKI, cardiac surgery–associated acute kidney injury; CTICU, cardiothoracic intensive care unit; DBF, direct breastfeeding; DOL, day of life; DORV, double outlet right ventricle; ECMO, extracorporeal membrane oxygenation; GA, gestational age; GT, gastrostomy tube; HAZ, height-for-age z score; HLHS, hypoplastic left heart syndrome; HM, human milk; HMF, human milk feeding; ICD-10, International Classification of Diseases, 10th Revision; ICU, intensive care unit; IQR, interquartile range; KDIGO, Kidney Disease: Improving Global Outcomes; LAZ, length-for-age z score; LS, long stay; n, number of participants; NEC, necrotizing enterocolitis; NEPHRON, Neonatal and Pediatric Heart and Renal Outcomes Network; NG, nasogastric (tube); NICU, neonatal intensive care unit; NIH, National Institutes of Health; NPC-QIC, National Pediatric Cardiology Quality Improvement Collaborative; NPO, nil per os (no oral intake); OR, operating room; PC4, Pediatric Cardiac Critical Care Consortium; PDA, patent ductus arteriosus; PGE1, prostaglandin E1; POD, postoperative day; PN, parenteral nutrition; S1P, stage 1 palliation; S2P, stage 2 palliation; SD, standard deviation; SS, short stay; SV, single ventricle; TF(s), trophic feed(s); TGA, transposition of the great arteries; ToF, tetralogy of Fallot; USA, United States of America; VIS, vasoactive–inotropic score; VSD, ventricular septal defect; WAZ, weight-for-age z score; oz, ounce(s)

Supplementary Table S4. Characteristics of included cohort and case-control studies

References:

1. Bertrandt RA, Gist K, Hasson D, Zang H, Reichle G, Krawczeski C, et al. Cardiac Surgery-Associated Acute Kidney Injury in Neonates Undergoing the Norwood Operation: Retrospective Analysis of the Multicenter Neonatal and Pediatric Heart and Renal Outcomes Network Dataset, 2015-2018. Pediatr Crit Care Med. 2024;25(5):e246-e57.
2. Dabbagh A, Miller S, McCulloch M, Rosenthal G, Conaway M, White S. Preoperative Oral Feeding in Infants with Congenital Heart Disease Within the First Month of Life is Associated with a Higher Likelihood of Freedom From Tube Feeding at Time of Postoperative Discharge. Pediatr Cardiol. 2025.
3. Elgersma KM, Wolfson J, Fulkerson JA, Georgieff MK, Looman WS, Spatz DL, et al. Human Milk Feeding and Direct Breastfeeding Improve Outcomes for Infants With Single Ventricle Congenital Heart Disease: Propensity Score-Matched Analysis of the NPC-QIC Registry. J Am Heart Assoc. 2023;12(17):e030756.
4. Menon SC, McCandless RT, Mack GK, Lambert LM, McFadden M, Williams RV, et al. Clinical outcomes and resource use for infants with hypoplastic left heart syndrome during bidirectional Glenn: summary from the Joint Council for Congenital Heart Disease National Pediatric Cardiology Quality Improvement Collaborative registry. Pediatr Cardiol. 2013;34(1):143-8.
5. Murray SE, Zimmerman D, Patel SS. Preoperative Feeding Fortification Among Infants with Congenital Heart Disease is Associated with Higher Growth Velocity in the First 30 Days Post-repair and Lower BMI Percentile for Age at 10 Years: A Retrospective Cohort Study. Pediatr Cardiol. 2025;46(2):394-400.
6. Scahill CJ, Graham EM, Atz AM, Bradley SM, Kavarana MN, Zyblewski SC. Preoperative Feeding Neonates With Cardiac Disease. World J Pediatr Congenit Heart Surg. 2017;8(1):62-8.
7. Toms R, Jackson KW, Dabal RJ, Reebals CH, Alten JA. Preoperative trophic feeds in neonates with hypoplastic left heart syndrome. Congenit Heart Dis. 2015;10(1):36-42.
8. Venna A, Reid K, Davis S, Gai J, d'Udekem Y, Clauss S. Preoperative Feeding in Single Ventricle Neonates is Predictive of Shorter Time to Goal Feed. Congenital Heart Disease. 2022;17(5):505-18.
9. Zacharias P, Blinci J, Shenoy R, Lee J, Singh Y. Impact of the Pre-Operative Standardized Nutritional Protocol in Infants with Congenital Heart Disease (CHD). J Cardiovasc Dev Dis. 2025;12(5).
10. Iliopoulos I, Burke R, Hannan R, Bolivar J, Cooper DS, Zafar F, et al. Preoperative Intubation and Lack of Enteral Nutrition are Associated with Prolonged Stay After Arterial Switch Operation. Pediatr Cardiol. 2016;37(6):1078-84.
