# Supplementary material for: Preoperative Nutrition-Based Interventions in Children Undergoing Cardiac Surgeries—A Systematic Review and Meta-Analysis
Source: Nutrients. 2026 Feb 6;18(3):544. doi: 10.3390/nu18030544 (PMC12899530; doi:10.3390/nu18030544)
Supplement: Supplementary file 1 [file nutrients-18-00544-s001.zip › 7. Suppl Table S5. NOS_cohort studies_11 Jan.pdf]

Supplementary Table S5. Assessment of risk of bias in cohort studies assessed using Newcastle-Ottawa Quality Assessment Scale

Quality Assessment Criteria	Criterion to be Fulfilled to Award Asterix (*)	Bertrandt 2024	Dabbagh 2024	Elgersma 2023	Menon 2013	Murray 2025	Scahill 2017	Toms 2015	Venna 2022	Zacharias 2025
		**Selection (4*maximum)**								
**Representativeness of the exposed cohort**	Truly representative of the average infant in the community or somewhat representative of the average infant in the community	*	*	*	*	-[1]	*	-[1]	*	-[1]
**Selection of the non-exposed cohort**	Drawn from the same community as the exposed cohort	*	*	*	*	*	*	*	*	-[2]
**Ascertainment of exposure**	Secure record or structured interview	-	*	*	*	*	*	*	*	*
**Demonstration that outcome of interest was not present at start of study**	Outcome of interest was not present at start of study? (Yes)	*	*	*	*	*	*	*	*	*
		**Comparability (2*maximum)**								
**Comparability of cohorts on the basis of the design or analysis**	If the study controls for at least one confounder (★) and if multiple confounders (★★)	**	**	**	**	-[3]	**	-[3]	**	-[3]
		**Outcome (3* maximum)**								
**Assessment of outcome**	Independent blind assessment or record linkage	*	*	*	*	*	*	*	*	*
**Was follow-up long enough for outcomes to occur**	Follow-up 'long enough'	*	*	*	*	*	*	*	*	*
**Adequacy of follow-up of cohorts**	Complete follow-up or small number lost to follow-up unlikely to introduce bias (less than 20% of number lost; or description provided of those lost)[4]	*	*	*	*	*	*	*	*	*

Supplementary Table S5. Assessment of risk of bias in cohort studies assessed using Newcastle-Ottawa Quality Assessment Scale

**TOTAL NOS**	**7**	**9**	**8**	**9**	**6**	**9**	**6**	**9**	**5**

Downgraded because of:

[1] limited representativeness of the exposed cohort due to the small sample size enrolled in a single center

[2] inappropriate selection of non-exposed cohort: mixed non-exposed group including historical (before development of the feeding protocol) and non-historical groups

[3] comparability: analysis adjusted for any potential co-founders was not performed

[4] given the predominantly retrospective design of the included studies, it was difficult to confirm that controls had no history of the outcome
