# Supplementary material for: Preoperative Nutrition-Based Interventions in Children Undergoing Cardiac Surgeries—A Systematic Review and Meta-Analysis
Source: Nutrients. 2026 Feb 6;18(3):544. doi: 10.3390/nu18030544 (PMC12899530; doi:10.3390/nu18030544)
Supplement: Supplementary file 1 [file nutrients-18-00544-s001.zip › 8. Suppl Table S6. NOS_case-control_19 Dec.pdf]

Supplementary Table S6. Assessment of risk of bias in case-control studies using Newcastle-Ottawa Quality Assessment Scale

Quality Assessment Criteria	Criterion to be Fulfilled to Award Star (★)	Illiapoulous 2016
		**Selection (4★ maximum)**
**Adequateness of case definition**	Case defined adequately, with independent validation<br>- children who underwent arterial switch operation with the transposition of grat arteries, with post-operative length of stay >14 days	★
**Representativeness of the cases**	Consecutive or obviously representative series of cases	★
**Selection of controls**	Community controls	-
**Definition of controls**	Length of stay >14 days compared with <7 days (endpoint)	★
		**Comparability (2★ maximum)**
**Comparability of cases and controls on the basis of the design or analysis**	If the study controls for at least one confounder (★) and if multiple confounders (★★)	-
		**Exposure (3★ maximum)**
**Assessment of exposure**	Independent blind structured interview or secure record as a source	★
**Same method of ascertainment for cases and controls**	(yes)	★
**Non-response rate**	Same rate for both groups	-
**TOTAL**		5
