# Supplementary material for: Preoperative Nutrition-Based Interventions in Children Undergoing Cardiac Surgeries—A Systematic Review and Meta-Analysis
Source: Nutrients. 2026 Feb 6;18(3):544. doi: 10.3390/nu18030544 (PMC12899530; doi:10.3390/nu18030544)
Supplement: Supplementary file 1 [file nutrients-18-00544-s001.zip › 9. Suppl Table S7. Elgeresma.pdf]

Supplementary Table S7. Summary of findings from study by Elegeresma et al.

Exposure	Summary of findings
Preoperative exclusive human milk feeding	Preoperative exclusive any human milk (HM) (maternal or donor) feeding was associated with a shorter length of stage 1 palliation hospital stay (OR=0.87, 0.78–0.98), a decreased risk of preoperative NEC (OR=0.37, 95% CI=0.17–0.84, n=1265). However, no difference in all-cause mortality between surgery and 1 year of age in infants fed exclusively with human milk preoperatively.
Preoperatively exclusive human milk feeding and any human milk feeding at discharge	Preoperative exclusive HM feeding and any human milk feeding at discharge was associated with shorter length of stage 1 palliation hospital stay discharge-(OR=0.75, 95% CI, 0.66 to 0.86), and lower risk of postoperative NEC (OR=0.28, 95% CI, 0.15 to 0.5) and any postoperative infection-related complications (OR=0.48, 95%CI, 0.25 to 0.91), and lower risk of postoperative sepsis (OR=0.29, 95% CI, 0.13 to 0.65; n=1067). However, no associations with time to achieving full feeds by infants after surgery and risk of all-cause mortality were observed.
Any preoperative direct breastfeeding and at discharge	Any preoperative direct breastfeeding and at discharge was associated with shorter hospital length of stay (RR=0.77, 95% CI, 0.66 to 0.9, n=1106) and lower risk of postoperative sepsis (0 vs 6.6%, OR=0). However, no associations with risk of preoperative NEC, any postoperative infection-related complications, and time to achieving full feeds by infants after surgery, and mortality were observed.
Breastfeeding and length of stage 2 palliation hospital stay	Infants receiving any human milk (RR=0.82, 0.69–0.97), fed exclusively with human milk (RR=0.75, 95% CI, 0.57 to 0.99), and with any direct breastfeeding (RR=0.71, 95%CI, 0.57 to 0.89) had shorter postoperative length of stage 2 palliation hospital stay.
